# Supplementary figures and images for: Diversity matters in wheat mixtures: A genomic survey of the impact of genetic diversity on the performance of 12 way durum wheat mixtures grown in two contrasted and controlled environments
Source: PLoS One. 2022 Dec 9;17(12):e0276223. doi: 10.1371/journal.pone.0276223 (PMC9733896; doi:10.1371/journal.pone.0276223)

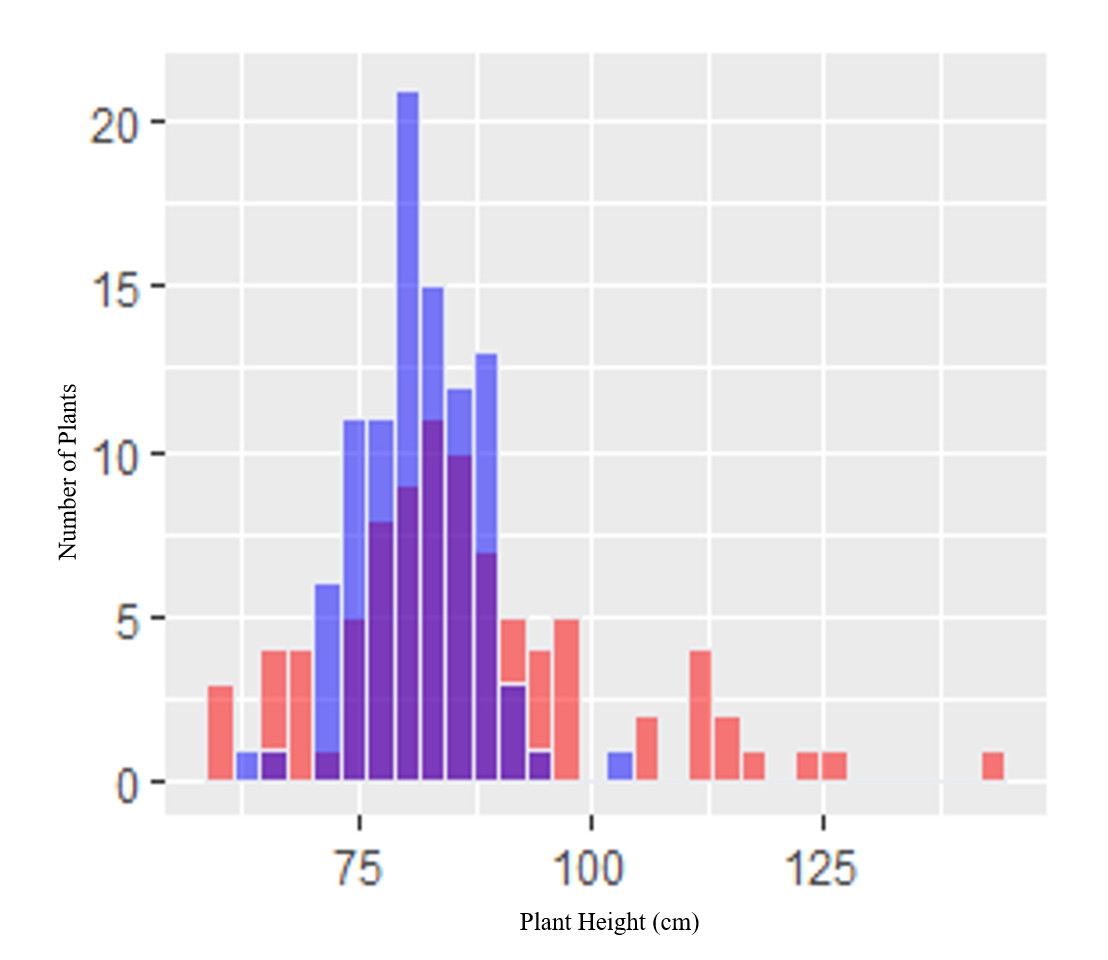

Supplement: S1 Fig — Red bars: 180 lines extracted from EPOs. Blue bars: 96 selected EPOs for the experiment, with uniform plant height. (TIF) [file pone.0276223.s001.tif]

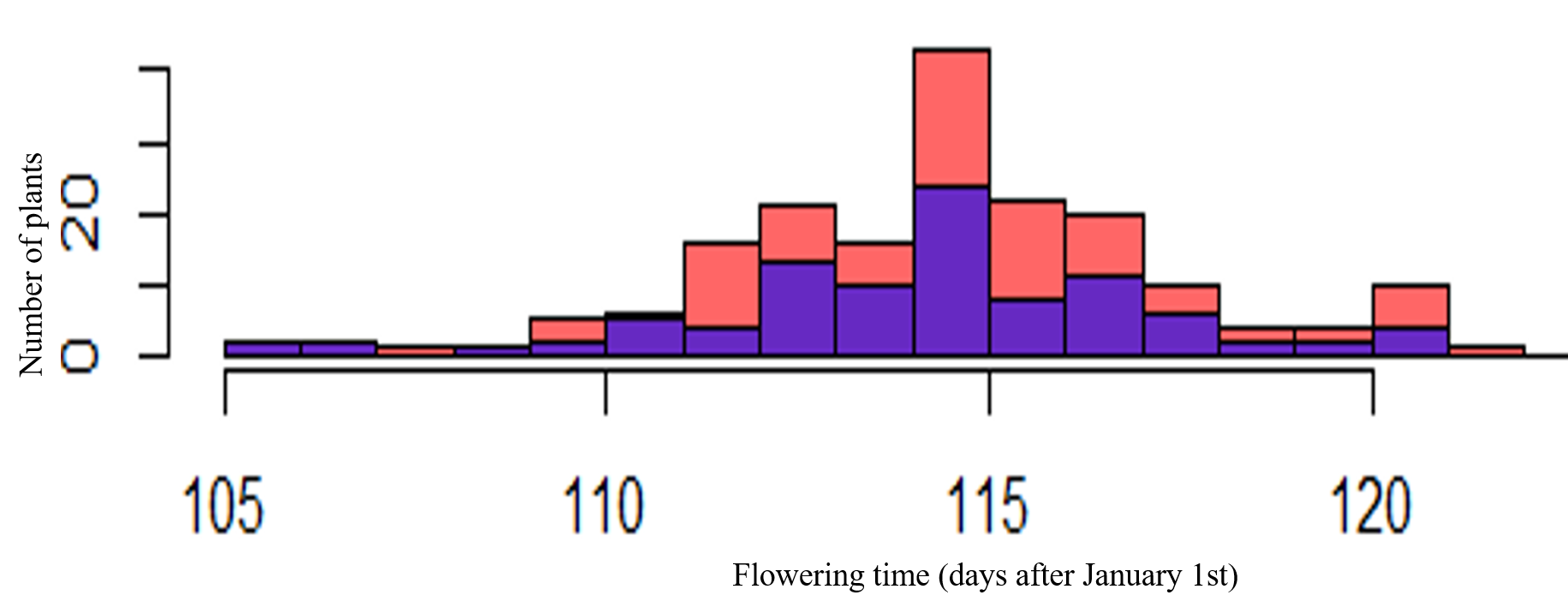

Supplement: S2 Fig — Red bars: 180 lines extracted from EPOs. Blue bars: 96 selected EPOs for the experiment, with uniform plant height. (TIF) [file pone.0276223.s002.tif]

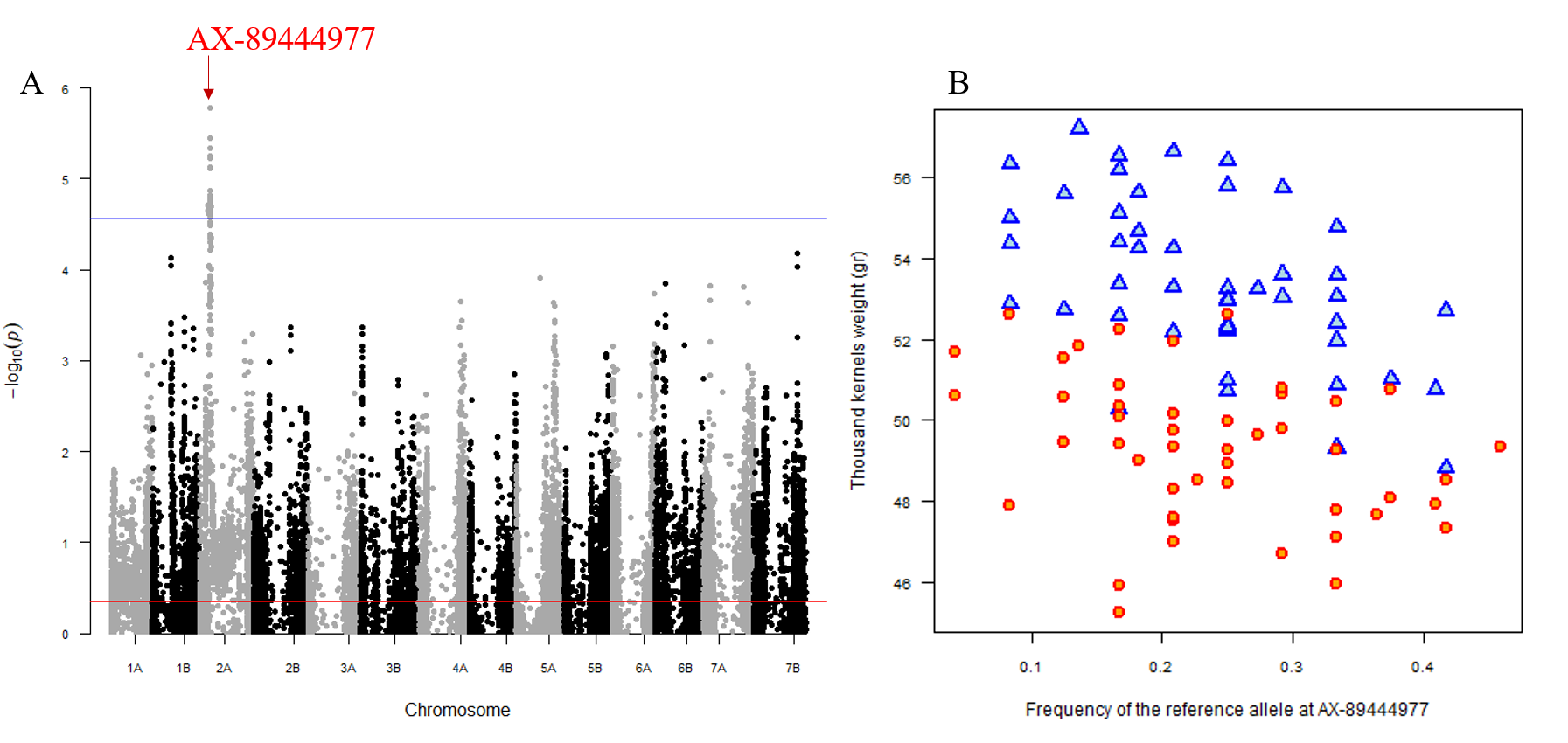

Supplement: S3 Fig — (A) Manhattan plot of the GWFA of TKW and 96k SNPs distributed along the durum wheat genome. The solid blue line represents the Family-Wise Error Rate (FWER) of 5% computed with the Galwey method (with a value of 4.55). (B) Relation between the allelic frequency of the peak SNP “AX-89444977” and TKW. Red points corresponds to plots under controlled water deficit conditions. Black points corresponds to plots under rainy conditions. (TIF) [file pone.0276223.s003.tif]
