# Supplementary material for: Diversity matters in wheat mixtures: A genomic survey of the impact of genetic diversity on the performance of 12 way durum wheat mixtures grown in two contrasted and controlled environments
Source: PLoS One. 2022 Dec 9;17(12):e0276223. doi: 10.1371/journal.pone.0276223 (PMC9733896; doi:10.1371/journal.pone.0276223)
Supplement: S1 Table — SpL: Spike Length in cm, TKW: Thousand Kernels weight in g, SNP: Single Nucleotide Polymorphism, Peak: physical position of the SNP on the chromosome in base pairs (bp), Lower bound: the minimal physical position that can have the SNP on the chromosome, Upper bound: the maximal physical position that can have the SNP on the chromosome, Chr: chromosome, QTL: Quantitative Trait Loci. The threshold of GWFA–log10(PValue) is 4.46. When GWFA–log10(PValue) is > 4.46 the association between the trait and the QTL is significant, and vice versa. * Significant–log10(PValue). (DOCX) [file pone.0276223.s004.docx]

**S1 Table**. *SNPs having significant associations between their allelic frequencies and the yield and others traits, with their –log10(PValue) and their positions on the chromosomes.*

*SpL: Spike Length in cm, TKW: Thousand Kernels weight in g, SNP: Single Nucleotide Polymorphism, Peak: physical position of the SNP on the chromosome in base pairs (bp), Lower bound: the minimal physical position that can have the SNP on the chromosome, Upper bound: the maximal physical position that can have the SNP on the chromosome, Chr: chromosome, QTL: Quantitative Trait Loci. The threshold of GWFA –log10(PValue) is 4.46. When GWFA –log10(PValue) is > 4.46 the association between the trait and the QTL is significant, and vice versa. * Significant –log10(PValue).*

| Trait | SNP | Lower bound | Peak | Upper bound | Chr | GWFA  -log10 (PValue) |
| --- | --- | --- | --- | --- | --- | --- |
| SpL | AX-89472110 | 44996650 | 44996650 | 44996650 | 1B | 4.79* |
|  | AX-89508436 | 617875916 | 710423213 | 714336717 | 2B | 4.63* |
|  | AX-89323384 | 606320622 | 607002696 | 607755271 | 4B | 4.96* |
|  | AX-89310738 | 16905847 | 68943239 | 325999950 | 5A | 4.66* |
|  | AX-89442901 | 411493178 | 411817387 | 412113431 | 5A | 5.22* |
| TKW | AX-89444977 | 147006948 | 152170092 | 153760309 | 2A | 5.77* |
