# Supplementary material for: Diversity matters in wheat mixtures: A genomic survey of the impact of genetic diversity on the performance of 12 way durum wheat mixtures grown in two contrasted and controlled environments
Source: PLoS One. 2022 Dec 9;17(12):e0276223. doi: 10.1371/journal.pone.0276223 (PMC9733896; doi:10.1371/journal.pone.0276223)
Supplement: S2 Table — SL: Stem length in cm, SpL: Spike Length in cm, NKS: Number of kernels per spike, NKM2: Number of kernels per m2, TKW: Thousand Kernels weight in g, Y: yield in gr/m², SNP: Single Nucleotide Polymorphism, Peak: physical position of the SNP on the chromosome in base pairs (bp), Lower bound: the minimal physical position that can have the SNP on the chromosome, Upper bound: the maximal physical position that can have the SNP on the chromosome, Chr: chromosome, QTL: Quantitative Trait Loci. The threshold of GWFA–log10(PValue) is 4.46. * Significant test. (DOCX) [file pone.0276223.s005.docx]

**S2 Table**. *QTLs having significant associations between their genetic diversity, the yield, and its components with the sign of the effect of their genetic diversity on each trait and their positions on chromosomes.*

*SL: Stem length in cm, SpL: Spike Length in cm, NKS: Number of kernels per spike, NKM2: Number of kernels per m^2^, TKW: Thousand Kernels weight in g, Y: yield in gr/m², SNP: Single Nucleotide Polymorphism, Peak: physical position of the SNP on the chromosome in base pairs (bp), Lower bound: the minimal physical position that can have the SNP on the chromosome, Upper bound: the maximal physical position that can have the SNP on the chromosome, Chr: chromosome, QTL: Quantitative Trait Loci. The threshold of GWDA –log10(PValue) is 4.46. * Significant test.*

| Trait | QTL | Lower bound | Peak | Upper bound | Chr | -log10 (PValue) | correlation with the trait (r) |
| --- | --- | --- | --- | --- | --- | --- | --- |
| HE_SL | AX-89416479 | 11794481 | 11808645 | 11808645 | 1A | 7.08_*_ | - 0.3 |
|  | AX-89591327 | 361874519 | 362238969 | 362238969 | 1A | 7.12_*_ | 0.3 |
|  | AX-89340128 | 15527946 | 17053574 | 16305107 | 1B | 6.19_*_ | 0.29 |
|  | AX-89348642 | 770248281 | 770248281 | 770248281 | 2A | 5.46_*_ | 0.26 |
|  | AX-89620480 | 146656465 | 150255445 | 153316716 | 2B | 6.17_*_ | 0.29 |
|  | AX-89526412 | 660956135 | 717809613 | 717809613 | 3A | 5.31_*_ | - 0.26 |
|  | AX-89697091 | 2913283 | 3070773 | 3285242 | 3A | 4.58_*_ | 0.23 |
|  | AX-89683286 | 716643751 | 659335285 | 717793866 | 3B | 6.47_*_ | -0.29 |
|  | AX-89440212 | 643903733 | 644007153 | 644007223 | 4B | 5.29_*_ | - 0.26 |
|  | AX-89371643 | 409952442 | 639901758 | 639903255 | 5A | 5.2_*_ | 0.26 |
|  | AX-89347305 | 461088825 | 461267138 | 618792407 | 5B | 7.65_*_ | 0.34 |
|  | AX-89309919 | 1812031 | 1812031 | 1812031 | 6B | 4.62_*_ | - 0.24 |
|  | AX-89773823 | 661924005 | 704817816 | 705466664 | 7B | 5.58_*_ | 0.27 |
|  | AX-89566514 | 293588922 | 293588922 | 293588922 | Un | 5.27_*_ | 0.26 |
| HE_SpL | AX-89425185 | 535349495 | 535349425 | 535349844 | 1A | 4.61_*_ | 0.37 |
|  | AX-89678958 | 10606731 | 1283946 | 11040259 | 1A | 4.97_*_ | - 0.39 |
|  | AX-89746162 | 11015066 | 14323398 | 14323398 | 1A | 4.7_*_ | - 0 .37 |
|  | AX-89676059 | 4186235 | 4459123 | 4493159 | 3B | 4.61_*_ | 0.37 |
|  | AX-89678958 | 1283946 | 1283946 | 1283946 | Un | 4.78_*_ | - 0.39 |
| HE_NKS | AX-89672881 | 32910445 | 32945067 | 32946674 | 1B | 5.4_*_ | -0.4 |
| HE_NKM2 | AX-89411835 | 32910445 | 32910375 | 32946674 | 1B | 4.94_*_ | - 0.39 |
|  | AX-89732178 | 666681187 | 668006646 | 668923536 | 1B | 5.12_*_ | - 0.40 |
|  | AX-89749150 | 820857539 | 820905359 | 820857539 | 3B | 5.49_*_ | - 0.41 |
| HE_TKW | AX-89703701 | 652033799 | 652033799 | 652033799 | 2A | 6.49_*_ | 0.32 |
|  | AX-89372423 | 784387839 | 784387839 | 784415453 | 2B | 5.46_*_ | 0.32 |
|  | AX-89498977 | 15811643 | 16127638 | 16319054 | 3B | 4.63_*_ | 0.28 |
|  | AX-89377854 | 635479979 | 635479909 | 635479979 | 4A | 4.92_*_ | 0.29 |
|  | AX-89387670 | 705615769 | 706918956 | 707926720 | 7B | 5.41_*_ | 0.31 |
| HE_Y | AX-89732178 | 666681187 | 668006646 | 668923536 | 1B | 5.73_*_ | - 0.34 |
|  | AX-89749150 | 820857539 | 820905359 | 820857539 | 3B | 6.06_*_ | - 0.39 |
