# Supplementary material for: Diversity matters in wheat mixtures: A genomic survey of the impact of genetic diversity on the performance of 12 way durum wheat mixtures grown in two contrasted and controlled environments
Source: PLoS One. 2022 Dec 9;17(12):e0276223. doi: 10.1371/journal.pone.0276223 (PMC9733896; doi:10.1371/journal.pone.0276223)
Supplement: S3 Table — SpL: Spike Length in cm, TKW: Thousand Kernels weight in g, SNP: Single Nucleotide Polymorphism, Peak: physical position of the SNP on the chromosome in base pairs (bp), Lower bound: the minimal physical position that can have the SNP on the chromosome, Upper bound: the maximal physical position that can have the SNP on the chromosome, Chr: chromosome, QTL: Quantitative Trait Loci. The threshold of GWFA–log10(PValue) is 4.46. * Significant test. (DOCX) [file pone.0276223.s006.docx]

**S3 Table**. *QTLs found that their allelic frequency is associated to traits, with their positions on the svevo genome of durum wheat, their –log10 (PValue) and the traits associated to their co-localized QTLs.*

*SpL: Spike Length in cm, TKW: Thousand Kernels weight in g, SNP: Single Nucleotide Polymorphism, Peak: physical position of the SNP on the chromosome in base pairs (bp), Lower bound: the minimal physical position that can have the SNP on the chromosome, Upper bound: the maximal physical position that can have the SNP on the chromosome, Chr: chromosome, QTL: Quantitative Trait Loci. The threshold of GWFA –log10(PValue) is 4.46. * Significant test.*

| _Trait_ | _SNP_ | Lower bound | Peak | Upper bound | _Chr_ | _GWFA_  _-log10 (PValue)_ | _Name of traits co-localized in the reference_ | _Reference_ |
| --- | --- | --- | --- | --- | --- | --- | --- | --- |
| _F_SpL_ | _AX-89472110_ | _44996650_ | _44996650_ | _44996650_ | _1B_ | _4.79*_ | _Yield_ | (Soriano et al., 2017) |
|  |  |  |  |  |  |  | _SpL_ | Unpublished from (Soriano et al., 2021) |
|  |  |  |  |  |  |  | _TKW_ | (Peleg et al., 2011; Peng et al., 2003) |
|  |  |  |  |  |  |  | _Spikelets per spike_ | (Faris et al., 2014) |
|  |  |  |  |  |  |  | _Grain yield per spike_ | (Soriano et al., 2021) |
|  |  |  |  |  |  |  | _Kernels roundness_ | (Russo et al., 2014) |
|  |  |  |  |  |  |  | _Grain yield_ | (Soriano et al., 2021) |
|  |  |  |  |  |  |  | _Root characteristics_ | (Iannucci et al., 2017; Petrarulo et al., 2015) |
|  |  |  |  |  |  |  | _Plant height_ | (Maccaferri et al., 2008) |
|  |  |  |  |  |  |  | _Spikes per m2_ | (Soriano et al., 2017) |
|  | _AX-89508436_ | _617875916_ | _710423213_ | _714336717_ | _2B_ | _4.63*_ | _TKW_ | (Soriano et al., 2017) |
|  |  |  |  |  |  |  | _root characteristics_ | (Maccaferri et al., 2016; Peleg et al., 2011) |
|  | _AX-89323384_ | _606320622_ | _607002696_ | _607755271_ | _4B_ | _4.96*_ | _kernels per m2_ | (Soriano et al., 2017) |
|  |  |  |  |  |  |  | _Test weight_ | (Maccaferri et al., 2011) |
|  |  |  |  |  |  |  | _Grain Yield_ | (Patil et al., 2013b) |
|  |  |  |  |  |  |  | _Spike density_ | Unpublished from (Soriano et al., 2021) |
|  | _AX-89310738_ | _16905847_ | _68943239_ | _325999950_ | _5A_ | _4.66*_ | _TKW_ | (Kidane et al., 2017) |
|  | _AX-89442901_ | _411493178_ | _411817387_ | _412113431_ | _5A_ | _5.22*_ | _Spikelets per spike_ | (Soriano et al., 2021) |
|  |  |  |  |  |  |  | _Kernels per spike_ | (Peng et al., 2003) |
|  |  |  |  |  |  |  | _kernels per spikelet_ | (Peng et al., 2003) |
|  |  |  |  |  |  |  | _Spike width_ | Unpublished from (Soriano et al., 2021) |
| _F_TKW_ | AX-89444977 | 147006948 | 152170092 | 153760309 | _2A_ | _4.6*_ | _Heading date_ | (Maccaferri et al., 2011) |
|  |  |  |  |  |  |  | _Gluten strength_ | (Fiedler et al., 2017) |
|  |  |  |  |  |  |  | _Semolina yield_ | (Colasuonno et al., 2017) |
|  |  |  |  |  |  |  | _Test weight_ | (Canè et al., 2014) |
