# Supplementary material for: Diversity matters in wheat mixtures: A genomic survey of the impact of genetic diversity on the performance of 12 way durum wheat mixtures grown in two contrasted and controlled environments
Source: PLoS One. 2022 Dec 9;17(12):e0276223. doi: 10.1371/journal.pone.0276223 (PMC9733896; doi:10.1371/journal.pone.0276223)
Supplement: S4 Table — SpL: Spike Length in cm, NKS: Number of kernels per spike, NKM2: Number of kernels per m2, TKW: Thousand Kernels weight in g, SNP: Single Nucleotide Polymorphism, Peak: physical position of the SNP on the chromosome in base pairs (bp), Lower bound: the minimal physical position that can have the SNP on the chromosome, Upper bound: the maximal physical position that can have the SNP on the chromosome, Chr: chromosome, QTL: Quantitative Trait Loci. The threshold of GWDA–log10(PValue) is 4.46. The threshold of HE vs F test–log10(PValue) is 2.78. * Significant test. (DOCX) [file pone.0276223.s007.docx]

**S4 Table**. *QTLs found that their genetic diversity is associated to traits, with their positions on the svevo genome of durum wheat, their –log 10 (PVal) and the traits associated to their co-localized QTLs.*

*SpL: Spike Length in cm, NKS: Number of kernels per spike, NKM2: Number of kernels per m^2^, TKW: Thousand Kernels weight in g, SNP: Single Nucleotide Polymorphism, Peak: physical position of the SNP on the chromosome in base pairs (bp), Lower bound: the minimal physical position that can have the SNP on the chromosome, Upper bound: the maximal physical position that can have the SNP on the chromosome, Chr: chromosome, QTL: Quantitative Trait Loci.*

*The threshold of GWDA –log10(PValue) is 4.46. When GWDA –log10(PValue) is > 4.46 the association between the trait and the QTL is significant, and vice versa. The threshold of HE vs F test –log10(PValue) is 2.78. * Significant test.*

| Trait | SNP | Lower bound | Peak | Upper bound | Chr | GWDA  -log10 (PValue) | Correlation with the trait (r) | HE vs. F test  -log10 (PValue) | Co-localization | Name of traits in reference | Reference |
| --- | --- | --- | --- | --- | --- | --- | --- | --- | --- | --- | --- |
| SpL | AX-89676059 | 4186235 | 4459123 | 4493159 | 3B | 4.61* | 0.37 | 3.21* | Oui | Grain yield | (Mengistu et al., 2016) |
|  |  |  |  |  |  |  |  |  |  | Heading date | (Maccaferri et al., 2014) |
|  |  |  |  |  |  |  |  |  |  | Average root length | (Maccaferri et al., 2016) |
| NKS | AX-89672881 | 32910445 | 32945067 | 32946674 | 1B | 5.41* | -0.41 | 4.99* | Yes | GY | (Mengistu et al., 2016) |
|  |  |  |  |  |  |  |  |  | Yes | TW | (Canè et al., 2014) |
|  |  |  |  |  |  |  |  |  | Yes | TKW | (Peleg et al., 2011) |
| NKM2 | AX-89411835 | 32910445 | 32910375 | 32946674 | 1B | 4.94* | - 0.39 | 3.96* | Yes  Yes  Yes | Grain yield | (Mengistu et al., 2016) |
|  |  |  |  |  |  |  |  |  |  | Test Weight | (Canè et al., 2014) |
|  |  |  |  |  |  |  |  |  |  | TKW | (Peleg et al., 2011) |
| TKW | AX-89377854 | 635479979 | 635479909 | 635479979 | 4A | 4.92* | 0.29 | 3.1* | Oui | SpL: Spike length | Unpublished from (Soriano et al., 2021) |
|  |  |  |  |  |  |  |  |  |  | Grain yield | (Mengistu et al., 2016) |
|  |  |  |  |  |  |  |  |  |  | Root characteristics | (Maccaferri et al., 2016) |
|  |  |  |  |  |  |  |  |  |  | Plant height | (Mengistu et al., 2016) |
|  |  |  |  |  |  |  |  |  |  | Biomass | (Mengistu et al., 2016) |
