# Supplementary material for: Diversity matters in wheat mixtures: A genomic survey of the impact of genetic diversity on the performance of 12 way durum wheat mixtures grown in two contrasted and controlled environments
Source: PLoS One. 2022 Dec 9;17(12):e0276223. doi: 10.1371/journal.pone.0276223 (PMC9733896; doi:10.1371/journal.pone.0276223)
Supplement: Attachment — Submitted filename: PONE-D-21-35051_reviewed.pdf [file pone.0276223.s008.pdf]

PLOS ONE

Diversity matters in wheat mixtures: a genomic survey of the impact of genetic diversity on the performance of 12 way durum wheat mixtures grown in two constrasted and controlled environments.

--Manuscript Draft--

**Manuscript Number:**	PONE-D-21-35051
**Article Type:**	Research Article
**Full Title:**	Diversity matters in wheat mixtures: a genomic survey of the impact of genetic diversity on the performance of 12 way durum wheat mixtures grown in two constrasted and controlled environments.
**Short Title:**	Diversity in durum mixtures matters.
**Corresponding Author:**	Pauline Alsabbagh<br>Montpellier SupAgro<br>MONTPELLIER, languedoc FRANCE
**Keywords:**	durum wheat; genetics; mixtures; diversity; kin selection; QTLs; drought; stress gradient; association genetics; GWAS; yield.
**Abstract:**	In ecology, an increase in genetic diversity within a community in natural ecosystems increases its productivity, while in evolutionary biology, kinship selection predicts that relatedness on social traits improves fitness. Varietal mixtures, where different genotypes are grown together, show contrasting results, especially for grain yield where both positive and negative effects of mixtures have been reported. To understand the effect of diversity on field performance, we grew 96 independent mixtures each composed with 12 durum wheat (Triticum turgidum ssp. durum Thel.) inbred lines, under two contrasting environmental conditions. Using dense genotyping, we imputed allelic frequencies and a genetic diversity index on more than 96000 loci for each mixture. We then analyzed the effect of genetic diversity on agronomic performance using a genome-wide approach. We explored the stress gradient hypothesis, which proposes that the greater the stress, the more beneficial the effect of diversity on mixture performance.
**Order of Authors:**	Pauline Alsabbagh
	Laurène Gay
	Michel Colombo
	Aline Rocher
	Vincent Allard
	Jacques L. David
**Additional Information:**	
**Question**	**Response**
**Financial Disclosure**<br><br>Enter a financial disclosure statement that describes the sources of funding for the work included in this submission. Review the submission guidelines for detailed requirements. View published research articles from *PLOS ONE* for specific examples.<br><br>This statement is required for submission and **will appear in the published article** if	JD was funded by the EU Horizon2020 SOLACE grant number 727247.<br>PA recieved a grant from "l'Association de spécialisation et d'orientation scientifique, Liban".

the submission is accepted. Please make sure it is accurate.<br><br>**Unfunded studies**<br>Enter: *The author(s) received no specific funding for this work.*<br><br>**Funded studies**<br>Enter a statement with the following details:<br>• Initials of the authors who received each award<br>• Grant numbers awarded to each author<br>• The full name of each funder<br>• URL of each funder website<br>• Did the sponsors or funders play any role in the study design, data collection and analysis, decision to publish, or preparation of the manuscript?<br>• **NO** - Include this sentence at the end of your statement: *The funders had no role in study design, data collection and analysis, decision to publish, or preparation of the manuscript.*<br>• **YES** - Specify the role(s) played.<br><br>* typeset	
**Competing Interests**<br><br>Use the instructions below to enter a competing interest statement for this submission. On behalf of all authors, disclose any competing interests that could be perceived to bias this work—acknowledging all financial support and any other relevant financial or non-financial competing interests.<br><br>This statement is required for submission and **will appear in the published article** if the submission is accepted. Please make sure it is accurate and that any funding sources listed in your Funding Information later in the submission form are also declared in your Financial Disclosure statement.<br><br>View published research articles from *PLOS ONE* for specific examples.	The authors declare no competing interest.

**NO authors have competing interests**<br><br>Enter: *The authors have declared that no competing interests exist.*<br><br>**Authors with competing interests**<br><br>Enter competing interest details beginning with this statement:<br><br>*I have read the journal's policy and the authors of this manuscript have the following competing interests: [insert competing interests here]*<br><br>* typeset	
**Ethics Statement**<br><br>Enter an ethics statement for this submission. This statement is required if the study involved:<br><br>• Human participants<br>• Human specimens or tissue<br>• Vertebrate animals or cephalopods<br>• Vertebrate embryos or tissues<br>• Field research<br><br>Write "N/A" if the submission does not require an ethics statement.<br><br>General guidance is provided below. Consult the submission guidelines for detailed instructions. **Make sure that all information entered here is included in the Methods section of the manuscript.**	N/A

**Format for specific study types**<br><br>**Human Subject Research (involving human participants and/or tissue)**<br>• Give the name of the institutional review board or ethics committee that approved the study<br>• Include the approval number and/or a statement indicating approval of this research<br>• Indicate the form of consent obtained (written/oral) or the reason that consent was not obtained (e.g. the data were analyzed anonymously)<br><br>**Animal Research (involving vertebrate animals, embryos or tissues)**<br>• Provide the name of the Institutional Animal Care and Use Committee (IACUC) or other relevant ethics board that reviewed the study protocol, and indicate whether they approved this research or granted a formal waiver of ethical approval<br>• Include an approval number if one was obtained<br>• If the study involved *non-human primates*, add *additional details* about animal welfare and steps taken to ameliorate suffering<br>• If anesthesia, euthanasia, or any kind of animal sacrifice is part of the study, include briefly which substances and/or methods were applied<br><br>**Field Research**<br><br>Include the following details if this study involves the collection of plant, animal, or other materials from a natural setting:<br>• Field permit number<br>• Name of the institution or relevant body that granted permission	
**Data Availability**<br><br>Authors are required to make all data underlying the findings described fully available, without restriction, and from the time of publication. PLOS allows rare exceptions to address legal and ethical concerns. See the PLOS Data Policy and FAQ for detailed information.	Yes - all data are fully available without restriction

A Data Availability Statement describing where the data can be found is required at submission. Your answers to this question constitute the Data Availability Statement and **will be published in the article**, if accepted.<br><br>**Important:** Stating 'data available on request from the author' is not sufficient. If your data are only available upon request, select 'No' for the first question and explain your exceptional situation in the text box.<br><br>Do the authors confirm that all data underlying the findings described in their manuscript are fully available without restriction?	
**Describe where the data may be found in full sentences. If you are copying our sample text, replace any instances of XXX with the appropriate details.**<br><br>• If the data are **held or will be held in a public repository**, include URLs, accession numbers or DOIs. If this information will only be available after acceptance, indicate this by ticking the box below. For example: *All XXX files are available from the XXX database (accession number(s) XXX, XXX.).*<br>• If the data are all contained **within the manuscript and/or Supporting Information files**, enter the following: *All relevant data are within the manuscript and its Supporting Information files.*<br>• If neither of these applies but you are able to provide **details of access elsewhere**, with or without limitations, please do so. For example:<br><br>*Data cannot be shared publicly because of [XXX]. Data are available from the XXX Institutional Data Access / Ethics Committee (contact via XXX) for researchers who meet the criteria for access to confidential data.*<br><br>*The data underlying the results presented in the study are available from (include the name of the third party*	All files are available from the database on the following link: https://data.inrae.fr/dataset.xhtml?persistentId=doi:10.15454/FG261F

and contact information or URL).<br>• This text is appropriate if the data are owned by a third party and authors do not have permission to share the data.<br><br>* typeset	
Additional data availability information:	

Powered by Editorial Manager® and ProduXion Manager® from Aries Systems Corporation

Manuscript

Click here to access/download;Manuscript;Alsabbagh_Manuscript11022021

Diversity matters in wheat mixtures: a genomic survey of the impact of genetic diversity on the performance of 12 way durum wheat mixtures grown in two constrasted and controlled environments.

Pauline Alsabbagh[1], Laurène Gay[1], Michel Colombo[1], Aline Rocher[1], Vincent Allard[2], Jacques L. David[1]

Corresponding author: jacques.david@supagro.fr

1 UMR AGAP, Université Montpellier, CIRAD, INRAE, Institut Agro Montpellier, Montpellier, France, 2 UMR GDEC, INRAE, Université Clermont Auvergne, Clermont-Ferrand, France.

Author contributions

PA produced morphological data, analysed data and wrote the paper, LG contributed substantially to the paper writing and discussions, AR was in charge of assembling the mixtures, MC contributed to the data analysis, VA had seminal ideas, hosted the experiment, produced data and contributed substantially to the writing, JD designed and supervised the project, analysed data and contributed to the writing,

Order of authors

1 Pauline Alsabbagh

2 Laurène Gay

3 Michel Colombo

4 Aline Rocher

5 Vincent Allard

6 Jacques L. David

Abstract:

In ecology, an increase in genetic diversity within a community in natural ecosystems increases its productivity, while in evolutionary biology, kinship selection predicts that relatedness on social traits improves fitness. Varietal mixtures, where different genotypes are grown together, show contrasting results, especially for grain yield where both positive and negative effects of mixtures have been reported. To understand the effect of diversity on field performance, we grew 96 independent mixtures each composed with 12 durum wheat (Triticum turgidum ssp. durum Thel.) inbred lines, under two contrasting environmental conditions. Using dense genotyping, we imputed allelic frequencies and a genetic diversity index on more than 96000 loci for each mixture. We then analyzed the effect of genetic diversity on agronomic performance using a genome-wide approach. We explored the stress gradient hypothesis, which proposes that the greater the stress, the more beneficial the effect of diversity on mixture performance.

We found that diversity on average had a negative effect on yield and its components while it was beneficial on grain weight. There was little support for the stress gradient theory. We discuss how to use genomic data to improve the assembly of varietal mixtures.

Introduction:

Maintaining crop yields will become increasingly challenging in the future due to the double constraint of an increased climate variability induced by climate change and a socio-economic context where both mineral inputs and phytochemicals use will tend to decrease [1–5]. For example, French wheat productivity measured in farms has reached a plateau since the 1990's [1,6,7], while the rate of genetic progress measured in non-limiting environments in experimental stations remained constant [4]. This strongly suggests that the genetic progress barely compensates for the progressive degradation of the pedo-climatic potential faced by farmers in real fields.

While successful genetic progress led to dramatic yield increases during the XXth century for many major crop species in intensive agricultural systems, this breeding strategy also contributed to an important decrease of the genetic diversity both within and between fields [8,9]. During the XXth century, it is indeed estimated that 75% of crop diversity vanished from the farmers' fields at a global scale [10]. Reintroducing higher levels of diversity in cultivated fields has been proposed as a putative solution to stabilize production and restore other ecological services [11–13], in particular within the context of sustainable agriculture [14–17]. Besides strategies to associate several crop species that received much attention, intra-specific genetic diversity has also been shown to play an important role for ecosystem functioning and stability [18,19]. In particular, varietal mixtures have been proposed as a tool to increase crop genetic diversity without the need of extensive breeding efforts [20] by assembling several varieties of the same species within the same field[3,5,21–23]. In France, varietal mixtures are under a renewed attention by farmers and the practice rose from below 2% of the wheat surface in 2010 to 8% in 2018 [24] mainly due to their capacity to stabilize yield over years [24,25].

When the yield of mixtures is explicitly tested in comparison with the average yield of their individual components grown in monoculture, the calculation of the mixture over-yielding is then possible [26]. Meta-analysis showed that, on average, over-yielding ranges between 3.5% for wheat [27], 3.9% for wheat and barley [12] and 5.4% for crop varietal mixtures [17]. Nevertheless this over-yielding is highly variable both between mixtures differing in composition and between environments. Negative over-yielding values have been observed in some situations [12,17,28]. The variability observed in mixture performance can find conceptual grounds in ecological theories. First, the hypothesis of niche complementarity

proposes that niche differentiation between organisms (component varieties in the present example) optimizes the use of natural resources, leading to an enhanced productivity [29]. Facilitation between different cultivars [30] or barrier and dilution effects of pathogens [27], could also promote natural regulatory processes within the mixture [24] by reducing negative density-dependent effects of enemies [31] and improve the performance of the mixture. Oppositely, negative effects of diversity can be explained by the kin selection theory. This hypothesis states that communities with higher genetic relatedness on some “social” traits should be more fit than genetically unrelated and diverse communities for these traits [32,33]. Mixture performance is also highly variable between environments, notably according to the environmental stress intensity. The ecological mechanisms acting in diverse communities, such as varietal mixtures could be less effective under optimal condition, in absence of biotic or abiotic stress than under stress [34–38]. Under this assumption, diverse communities are able to maintain functioning during increased environmental stress due to species redundancy that provides a greater guarantee to maintain functioning due to the tolerant species within the mixture, compensating for the failure of sensitive species [39]. The intensity of facilitation and competition between plants thus varies along environmental gradients with clear positive interactions dominating under stressful conditions [34]. In the specific case of varietal mixtures, such an effect has been observed through a clear over-yielding increase along a biotic stress gradient [27]. However, in that study, abiotic stress variations did not produce any significant effect on over-yielding.

Given this observed high variability, understanding the mechanisms underlining mixture performances is necessary to help farmers design efficient mixtures. If a few assembly rules have been proposed in the specific context of disease control [25], the identification of key criteria for mixture assembly is still necessary [40,41], in particular to design mixtures adapted to abiotic stresses. The recent progress of genomics and high-throughput genotyping that give access to allelic variations along the genome at a moderate cost [42,43] opens a great opportunity to understand the effect of genetic diversity on mixture performance at the gene level. This could then be used to design criteria for mixture assembly. For example, a positive relationship between the diversity over a single 310 kb genomic region was found associated with an increase of productivity of *Arabidopsis thaliana* two-component mixtures [44]. Identically, another study on mixtures of genotypes of Arabidopsis thaliana, showed a SNP on the chromosome 2A which has a higher size effect in polycultures than in monocultures [45]. Oppositely, genetic diversity at a single locus on chr 6B was negatively associated with grain

yield in two-component mixtures of durum wheat [46]. These two examples illustrate the complexity of the mechanisms through which within-field plant genetic diversity in particular, may be beneficial or detrimental to crop performance. These two studies, like the vast majority of the studies that tackle the question of varietal mixtures, are based on two-way balanced mixtures. Yet, the mixtures with highest interest for agronomical perspectives will likely be more complex, since the number of species has been identified as a key criterion affecting ecosystem performances [47].

To go beyond the very large combinatory of multiple ways mixtures, we propose here to work on complex mixtures with high genetic diversity, with an explicit focus on the effects of diversity at a large number of loci on mixture performance. We report the results of an experiment where we built 96 mixtures, each made up of 12 independent durum wheat lines (*Triticum turgidium subsp. durum*) chosen within a panel of 96 lines. Given the complexity of the experimental design, individual lines were not observed in monoculture and over-yielding could not be computed. The objective of the study was not to demonstrate the agronomic interest of mixtures that is now aknowledged, but rather to explore a large range of allelic variation on several thousands of markers and seek for association between the phenotypic values of major agronomical traits and within-mixture genetic diversity. To this aim we extended the classical GWAS approach to a Genome Wide Diversity Association studies (GWDAS). Furthermore, in order to test the stress-gradient hypothesis, these associations were studied in two contrasted conditions for water availability. In durum wheat, drought is one of the main causes of yield losses that can vary from 10 to 80% depending on the year, in the Mediterranean region [48]. We therefore explored how the effects of genetic diversity were affected by drought, a major abiotic stress, using the Pheno3C INRAE phenotyping platform at INRAE Clermont-Ferrand.

Materials and methods:

Mixtures design

Ninety-six inbred lines of durum wheat (*Triticum triticum durum* Th.), were chosen from a set of 180 inbred lines, derived from an evolutiona[illegible]re-breeding population, hereafter EPO, developed at INRAE Montpellier, France [49–51][49–51]. These lines have been described for a large number of traits, and exhibit high phenotypic variability for both above- and belowground traits [49], and harbour interesting major resistances to foliar diseases [51]. We discarded extreme tall and short components to reduce the impact of an obvious and already well-described competition due to plant height for light and resources among lines within

plots [49,52,53]. Among the 180 lines extracted from EPO, we selected 96 lines to maximize homogeneity in stem height (90-110 cm, S1 Fig.) and restrict flowering date variation between lines to 5 days (S2. Fig).

Ninety-six (96) 12-way mixtures were prepared by assembling for each of them an even number of seeds (70) from twelve distinct durum wheat lines, drawn from the 96 selected genotypes. Together, the 96 mixtures represent an incomplete balanced design. No mixture had the same composition and each line was present in 12 mixtures with an independent set of neighbours. The design was realized by a homemade R script. The rationale was to sample mixtures iteratively, sampling at each step new mixtures in the remaining available lines and verifying the unicity of each mixture.

S1 Fig. The distribution of plant height of the 96 EPOs selected from the 180 lines of EPOs extracted for our experimentation. Red bars: 180 lines extracted from EPOs. Blue bars: 96 selected EPOs for the experiment, with uniform plant height.

S2 Fig. The distribution of earliness of the 96 EPOs selected from the 180 lines of EPOs extracted for our experimentation. Red bars: 180 lines extracted from EPOs. Blue bars: 96 selected EPOs for the experiment, with uniform plant height.

Field experiment

The 96 mixtures were sown in Clermont-Ferrand, France, at INRAE Crouël (45˚78' N, 3˚08' E, 401 m a.s.l), in October 2017 on the Phéno3C field-phenotyping platform (INRAE PHACC Experimental Unit). Phéno3C is installed on a browned fluvisol with predominantly clay-loam soil. The surface horizon has a pH of 8.04, a percentage of organic matter content of 3.5, and a depth of 90 cm, its water reserve is 260 mm. This platform offers unique conditions to contrast in the same site, controlled water deficit (CWD) and rainy (R) conditions, via a system of rain-out mobile shelters, to generate the required level of water stress. Mixtures were sown in 8-rows plots of 2.185 m^2 each (1.15 m width, 1.9 m length and 17 cm between rows), in four blocks in each R and CWD conditions (48 mixtures in each). The trial was managed following local agronomic practices. The sowing was realized on 15/11/2017, and the harvest on 03/07/2018 for CWD and on 10/07/2018 for R plots. The 48 mixtures under CWD were covered by automatic rain-out from 02/02/2018 to create a controlled water deficit. The 48 mixtures under R, were maintained under the local climate and no irrigation was needed.

Measurement of soil water content

To measure the soil volumetric water content (SWC), sensors of CS 655 (Cambell Scientific, Logan, UT, USA) were installed at four depth (10, 35, 50 and 75 cm) at three locations per plot to record the volume hourly. Total soil water content was calculated by integrating sensor values up to 100 cm depth (average soil depth of the plots). Average soil field capacity and available water capacity are respectively 570 and 280 mm. All other environmental conditions were identical between the two regimes.

Phenotypic data

Traits recorded at the plot level

Heading date (HD) was measured when 50% of the spikes headed. The number of spikes per meter square (NSM2) was determined after anthesis, by manual counting of spikes on a 1 m long transect in the central row. After harvest, the yield of each mixture (Y) was computed as the raw weight of harvested kernels, measured at a 0% of humidity (humidity-meter, TM, Tripette and Renaud, France). It was corrected according to the number of some missing rows due to cropping aleas. Y is finally given in grams per m^2. The thousand Kernels Weight (TKW) was obtained from a representative sampling of 1200 kernels from the whole plot harvest after first division by half with a Seedburo divider followed by second division by 8 using a precision Retsch divider. A final count of 1200 kernels was done on a Contador device (Pfeuffe) and then precisely weighed. The Number of Kernels per meter square (NKM2) was calculated from Y and TKW as NKM2= 1000 x Y/ TKW. The number of kernels per spike (NKS) was calculated as NKS= 1000xY/(TKWxNSM2).

Traits recorded on 10-individual spikes per plot

Ten stems with fully developed spikes (stems with aborted spikes were discarded) were collected randomly from the central row in each mixture/plot before harvest. Stems were cut at the soil level. We measured the stem length (SL, cm) and the spike length (SpL, cm). Average values of these traits were then computed for each mixture.

Genotypic data

Genotypic data for the 96 EPO lines was obtained in a previous study using the high-throughput genotyping array TaBW280K [43], as detailed in Ballini (2020) [51].

The physical positions of the SNPs were estimated by blasting first on the reference genome SVEVO [54] and confirmed by linkage disequilibrium analysis. We only kept the SNPs for which we had a hit on the genome sequence by Basic Local Alignment Search Tool (BLAST). The genotyping data resulted in 96,562 polymorphic SNPs after filtering. Allele frequencies of SNPs in each of the 96 12-way mixtures were estimated assuming a balanced contribution from each of the 12 lines:

$$Fi,j = \frac{1}{12}\sum_{k=1}^{12} G_i^{k,j}$$

where Fi,j is the allele frequency of the ith SNP (from 1 to 96,562) in the jth mixture (j from 1 to 96) and $G_i^{k,j}$ is the genotype of the kth (from 1 to 12) line used in mixture j at locus i, and can be 2, 1 or 0. This assumes that allele frequencies in each mixture remained constant between the seed and adult plant stages. This is likely, given that all lines are homogeneous for their germination rate, since all the grains where produced in the same place, which eliminate the control of maternal effect and have homogeneity in competitive ability (mainly driven by plant height).

We measured genetic diversity of each mixture at each locus using the Nei's heterozygosity [55] as follows:

$$HE_{i,j} = 2F_{i,j}(1 - F_{i,j})$$

Because diversity is a function of allele frequency, it is highest when allele frequencies are balanced. There is therefore a risk to detect an effect of diversity that is in fact an effect of allele frequency, for example, when a favourable allele is rare. To avoid this confusion to test the effect of diversity, we removed the SNPs with rare alleles: we kept only SNPs for which the allele frequency spanned at least 0.5 around the median frequency of 0.5, i.e., we removed the SNPs for which the mixture with the lowest allele frequency still had an allele frequency above 0.5 ($\min(F_j) > 0.5$) and the SNPs with $\max(F_j) < 0.5$, as well as those with $\max(F_j) - \min(F_j) < 0.5$. The resulting dataset for diversity comprised 31,059 SNPs.

Statistical analysis

All analyses were performed on the software R. All scripts and data are available on https://data.inrae.fr/dataset.xhtml?persistentId=doi:10.15454/FG261F.

Preliminary analyses

To evaluate the impact of the two water regimes on each trait, a simple ANOVA declaring treatments and blocks was realized. To determine the relationship among traits and the influence of the water regime on these relations, we performed a matrix of Pearson correlation [56] on the whole data set (All treatments) declaring a treatment effect, and on the two water regime data sets separately.

Genome Wide Genetic Diversity Association (GWDA) and Genome Wide Frequency Association (GWFA)

The GWDA, respectively GWFA, analysis were build by regression analysis on HE, resp, F, including water regimes. We also explored the interaction between both genetic variables (F and HE) with the water regime in order to reveal potential "Genotype x Environment" effects. We included a block effect to account for the experimental design. This resulted in the following model(s) for each SNP:

$$P = \mu + Block_T + T + HE_k(F_k) + TxHE_k(TxF_k) + e,$$

where P is the phenotypic trait in each mixture, T the effect of the water regime (CWD or R), $Block_{/T}$ the block nested in the treatment T effect. HE_k (GWFA) and F_k (GWDA) are respectively the regression slopes of the genetic diversity, resp. the allele frequency at the kth SNP locus, and $TxHE_k$ (or resp. TxF_k) the interaction effect between the treatment and genetic diversity (or resp. allelic frequency). Treatments, diversity and interaction effects were assessed using a sequential type 1 test with the least square method. Effect were tested in the following order (Block, T, He_{k}, resp. F_k) and $TxHE_k$ (resp. TxF_k). P-values were –log10 transformed for clarity as in a GWAS study. Since HE is a second degree polynomial in F, (HE = 2F(1-F)), a linear regression based on HE may be overfitting a simple linear regression based on F. For each locus for which we found a significant association with HE, we compared the GWDA to the GWFA one by an anova test (thereafter called the "HE vs. F test"). Its significance threshold was determined by a Bonferonni correction to account for independent detection of multiple QTL.

For the QTLs found associated to the interaction between HE and the environment, we calculated the correlation between the allele frequency of the associated QTL and the trait in each environmental condition separately.

Significance threshold

The threshold for significant association, i.e., for declaring a HE-QTLs (with GWDA) and F-QTLs (with GWFA) was determined using the Galwey method [57]. Briefly, a matrix of linkage disequilibrium among polymorphic SNPs was used to estimate the effective number of independent tests. We found 1774 independent segregating chromosomes fragments through the method. A corrected Bonferroni threshold for was then 0.05/1774, giving a –log10(PValue) threshold of 4.46.

Quantitative trait Loci (QTL) boundaries

A method inspired from [58] was used to define QTL boundaries for each SNP showing a significant signal of association. For each chromosome and each trait, we computed the linkage disequilibrium (LD) among significant markers, using Hill and Robertson's R^2 estimator [59]. These LDs were square roots transformed to approximate a normally distributed random variable [60]. Then markers were clustered by LD blocks. Clustering was realized by average distance using a cutoff of 1-"critical R^2". Critical R^2 (R^2c) was defined as the 99.9th percentile of the distribution of unlinked R^2 computed between pairs of markers randomly sampled from different chromosomes. This threshold accounts for a risk of 0.1% to be in LD by chance. QTL boundaries were finally defined as the minimum and maximum map position of significant markers belonging to the same LD block. The most significant marker was used to represent QTL size effect and minor allele frequency (MAF). QTL of different traits were considered to overlap when they had at least one common significant marker and were located at a physical distance below one tenth of the total physical length of the chromosome, as presented in [58].

Co-localization of QTL with published QTL

Using the [Triticum turgidum Durum Wheat Svevo (RefSeq Rel. 1.0) - GrainGenes (usda.gov)] and the table of the meta-QTL analysis [61], our QTLs were co-located using their physical location in bp on the SVEVO genome of durum wheat using the interval defined beyond [54].

Global genomic approach of water regime impact

To study the impact of genetic diversity at the genome level, we aggregated the effects of diversity across loci for each water regime and for each trait. Only SNP markers fitting the conditions for HE computations (see Genotypic data) for both treatments were kept, only 13,093 were left. We first fitted the following model for each SNP and every trait in each treatment (CWD and R):

$$P = \mu + \beta_k HE_k + Block \quad + e,$$

where P is the response trait in each plot (from 1 to 48, either in R or in CWD), $\beta_k$is the slope of the partial regression of the genetic diversity (HE) at locus k (k from 1 to 13,093) on the trait, and Block are the blocks in the considered treatment.

We obtained for each SNP a pair of regression slopes for the effect of HE on the trait, both in R and in CWD. We counted the number of positive or negative HE slopes for each trait and each water treatment. We assessed whether diversity had a rather positive or negative effect on the trait by testing whether there was a significant difference in the number of positive and negative slopes by a Wilcoxon test. In a second step, we examined the stress gradient hypothesis (HE are has a more positive effect in stress environment, i.e., higher under CWD than under R) by testing whether the slopes under CWD were on average higher than under R, using a paired Wilcoxon test on the paired differences in slopes computed across treatments for each SNP.

Results

Water stress

The soil water reserve in the R plots never felt significantly below the limit of "easily accessible water" during the whole crop cycle leading to infer that plants in R condition did not experience water stress (Fig 1). In the CWD, from the 7th of April (booting stage) to the end of the plant cycle, the water reserve fell below the threshold (400 mm) of the easily accessible water and reached extremely low levels during the last part of the cycle

Fig 1. Dynamic of the cumulative rainfall from sowing and of the soil water reserve Pheno3C for the 2018 campaign, in the two treatments. Horizontal lines figure the soil water reserve (in mm) at Field Capacity Easily accessible water and permanent wilting point. Blue line is for plots under rainy conditions (not sheltered). Red line is for plots under controlled water deficit conditions (under shelter from the end of February).

Impact of controlled drought on phenotypic traits

CWD had a significant effect on all traits studied, except SpL (Table 1). Under stress, on average, plants flowered 5 days earlier. Plant morphology was affected by stress leading to a reduction of plant height by nearly 10%. In term of yield elaboration, water stress reduced significantly all yield components. The impact on TKW (-7.63%), NSM2 (-8%) and NKS (-

10.3%) was moderate but severe on NKM2 (-25%). These effects are in the expected direction since it is known that stress reduces performance.

Table 1. Average values in durum wheat mixtures between two water regimes for 8 traits.

*CWD: Controlled water deficit conditions, R: Rainy conditions, SL: Stem length in cm, SpL: Spike length in cm, NSM2: Number of spikes per m^2, NKS: Number of kernels per spike, NKM2: Number of kernels per m^2, TKW: Thousand kernel weight in g, Y: Yield in gr/m^2, HD: Heading date: days after 1/1/2018, Average Mean difference (%): the percentage of difference in the trait between the two conditions. The threshold of –log10(PValue) is 1.301. * Significant test.*

	Traits	R	CWD	Average Mean difference (%)	-log10 (PValue)
Average of 10 individual spikes	SL	74	67	-9.45	> 15.65*
	SpL	6.9	6.9	0	0.39
Plots value	NSM2	294	272	-8	1.6*
	NKS	34.69	31.09	-10.3	2*
	NKM2	10560.95	8441.714	-25	5.6*
	TKW	53.43	49.35	-7.63	15.21*
	Y	266,5	204	-24	10.10*
	HD	127	122	-4.75	>15.65*

Correlation between agronomic traits.

Under both conditions, Y was correlated to NKM2 and NSM2, and NKM2 was correlated to NSM2. However, water condition played a big role in traits relationships. For instance, plots having longer stem were more productive in the R condition (r(SL-NKM2)=+0.3, p-value= 0.03) but with no significant correlation under CWD (-0.12, p-val=0.4). Relationship between NKM2 and TKW was also affected by the water regime. Under water stress, the correlation that was not significant but was significantly negative (r = -0.43, p-value=0.002) under rainy conditions (Fig 2). This indicates that water stress induced a compromise between grain size and grain number.

Fig 2. Pairwise Pearson correlation coefficients (r) between functional traits for all the 48 mixtures (A) under controlled water deficit conditions and (B) the 48 mixtures under rain-fed conditions. The significant threshold has been set at 5%. Positive correlation are in blue and

negative in red. More the circle is big more the correlation is significant and vice versa. More the colour of the circle on the number is dark more the correlation is big and vice versa. Traits have been clustered according to the hclus procedure of the corrplot R package function.

SL: Stem length in cm, SpL: Spike length in cm, NSM2: Number of spikes per m2, NKS: Number of kernels per spike, NKM2: Number of kernels per m2, TKW: Thousand kernel weight in g, Y: Yield in gr/m².

Genome wide association studies

Associations with allelic frequencies (GWFA)

Six F-QTLs involving SNP frequencies were detected from 604 SNPs with –log10(PValue) higher than the threshold. Traits involved were SpL and TKW (S1 Table). SpL has the highest number of significant associations in GWFA (5 F-QTLs), while only one F-QTL was found for TKW (S1 Table). The F-QTL of TKW, located on the chromosome 2A had the most significant association with a –log10 (PValue) of 5.77 (S3 Fig and S1 Table).

S3 Fig. Genome wide Association between allelic frequencies and TKW on a set of 96 12 way mixture and 96,582 bi allelic SNP. (A) Manhattan plot of the GWFA of TKW and 96k SNPs distributed along the durum wheat genome. The solid blue line represents the Family-Wise Error Rate (FWER) of 5% computed with the Galwey method (with a value of 4.56). (B) Relation between the allelic frequency of the peak SNP "AX-89444977" and TKW. Red points corresponds to plots under controlled water deficit conditions. Black points corresponds to plots under rainy conditions.

S1 Table. SNPs having significant associations between their allelic frequencies and the yield and others traits, with their –log10(PValue) and their positions on the chromosomes.

SpL: Spike Length in cm, TKW: Thousand Kernels weight in g, SNP: Single Nucleotide Polymorphism, Peak: physical position of the SNP on the chromosome in base pairs (bp), Lower bound: the minimal physical position that can have the SNP on the chromosome, Upper bound: the maximal physical position that can have the SNP on the chromosome, Chr: chromosome, QTL: Quantitative Trait Loci.

*The threshold of GWFA –log10(PValue) is 4.46. When GWFA –log10(PValue) is > 4.46 the association between the trait and the QTL is significant, and vice versa. * Significant –log10(PValue).*

Genotype (F) x Environment

Pvalues of Treatment x Frequencies were rarely significant except for one F-QTL (AX-89757725, –log10(PValue) =5.14) associated with TKW, located at 546,024,925 bp, within the

interval [545,597,462; 547,234,231] on the chr 7B. For this QTL, the effect of F was low in R but had a strong effect in CWD. The frequency variation range for this F-QTL was not large. The increase in frequency was favourable under water stress conditions and neutral in rain-fed conditions.

Association with diversity (GWDA)

From the 31K SNPs considered, 211 showed a significant signal of association involving 6 traits. These 211 SNPs were grouped in 30 HE-QTLs on Y, NKS, NKM2, TKW, SL and SpL (S2 Table). It is striking to see that the number of traits involved in the associations with SNP diversity is higher than in GWFA. To ascertain that these associations with HE were not due to the overfitting of a simple linear regression based on F, we compared by the *anova* function of R, the HE and F models for the 30 HE-QTLs, (HE vs. F test) with a significance threshold of 0.001 (i.e., 0.05/30) or a –log10(PValue) = 2.78.

Four HE-QTLs were left significantly more associated with HE than with F (Table 2): one HE-QTL associated with the NKM2 (–log10(PValue(HE vs F)) = 3.96) on the chr 1B, with a negative effect of HE; one HE-QTL for the NKS (–log10(PValue(HE vs F))= 4.99) on the chr 1B with a negative effect (Fig 3), who is the same HE-QTL found for the NKM2; one HE-QTL for the TKW (–log10(PValue(HE vs F)) = 3.19) on the chr 4A, with a positive effect (Fig 4) and one HE-QTL for SpL (–log10(PValue(HE vs F)) = 3.21) on the chr 3B, with a positive effect. For the 26 others HE-QTLs, HE did not improve the model compared to F, even though no significant associations were detected with F. This may be due to a lack of power and the distribution of allele frequencies. This approach is certainly over-conservative, but it ensures that associations detected with HE had a reduced probability to be false positives due to overfitting.

Fig 3. Negative impact on NKM2 of genetic diversity of the SNP “AX-89411835” located on the chromosome 1B. (A) Manhattan plot reporting p-values (-log10 transformed) for the association tests between NKM2 and diversity at 31k SNPs distributed along the durum wheat genome (GWDA). The solid blue line represents the Family-Wise Error Rate (FWER) of 5% computed with the Galwey method. (B) Distribution of the allelic frequency of the SNP “AX-89411835” located on the chromosome 1B and NKM2. Red points corresponds to stressed plots. Black points corresponds to rain-fed plots. (C) Relation between the genetic diversity of the SNP “AX-89411835” and NKM2. Red points corresponds to stressed plots. Black points corresponds to rain-fed plots. The red line represents the slope of the regression.

Fig 4. Positive impact on thousand-kernel weight (TKW) of the genetic diversity of the associated SNP “AX-89377854” located on the chromosome 4A. (A) Manhattan plot reporting p-values (-log10 transformed) for the association tests between TKW and diversity at 31k SNPs distributed along the durum wheat genome (GWDA). The solid blue line represents the Family-Wise Error Rate (FWER) of 5% computed with the Galwey method. (B) Distribution of the allelic frequency of the SNP “AX-89377854” and TKW. Red points corresponds to plots under controlled water deficit conditions. Black points corresponds to plots under rain-fed conditions (C) Relation between the genetic diversity of the SNP “AX-89377854” and TKW. Red points corresponds to plots under controlled water deficit conditions. Black points corresponds to plots under rain-fed conditions. The red line represents the slope of the regression.

S2 Table: QTLs having significant associations between their genetic diversity, the yield, and its components with the sign of the effect of their genetic diversity on each trait and their positions on chromosomes.

*SL: Stem length in cm, SpL: Spike Length in cm, NKS: Number of kernels per spike, NKM2: Number of kernels per m^2, TKW: Thousand Kernels weight in g, Y: yield in gr/m², SNP: Single Nucleotide Polymorphism, Peak: physical position of the SNP on the chromosome in base pairs (bp), Lower bound: the minimal physical position that can have the SNP on the chromosome, Upper bound: the maximal physical position that can have the SNP on the chromosome, Chr: chromosome, QTL: Quantitative Trait Loci. The threshold of GWFA –log10(PValue) is 4.46. * Significant test.*

Table 2. HE-QTLS Significant associations between genetic diversity and traits.* Significant –log10 (PValue).

SpL: Spike Length in cm, NKS: Number of kernels per Spike, NKM2: Number of kernels per m^2, TKW: Thousand Kernel Weight, SNP: Single Nucleotide Polymorphism, Peak: physical position of the SNP on the chromosome in base pairs (bp), Lower bound: the minimal physical position that can have the SNP on the chromosome, Upper bound: the maximal physical position that can have the SNP on the chromosome, Chr: chromosome, QTL: Quantitative Trait Loci, r: value of the correlation between the genetic diversity of the QTL and the trait.

*The threshold of GWDA –log10(PValue) is 4.46. When GWDA –log10(PValue) is > 4.46 the association between the trait and the genetic diversity of the QTL is significant, and vice versa. The threshold of HE vs F test –log10(PValue) is 2.78. * Significant test.*

Trait	SNP	Lower bound	Peak	Upper bound	Chr	GWDA –log10(PValue)	HE vs F test –log10(PValue)	r

SpL	AX-89676059	4186235	4459123	4493159	3B	4.61*	3.21*	+ 0.37*
NKS	AX-89672881	32910445	32945067	32946674	1B	4.99*	4.99*	-0.41*
NKM2	AX-89411835	32910445	32910375	32946674	1B	4.94*	3.96*	- 0.39*
TKW	AX-89377854	635466248	635479909	635479979	4A	4.92*	3.1*	+ 0.29*

Genotype (HE) x Environment

For Y and NKM2, we found one common significant HE-QTL, located at 7,275,378 bp on the chr 2A with varying effect depending on the water conditions. HE had a non-significant negative effect under R, but a significant positive effect under CWD (Fig 5). This HE-QTL of the G x E interaction remained significant after the HE vs. F test for Y (–log10 (PValue) = 3) but not for NKM2 (-log10 (PValue) = 0.1).

Fig 5. Contrasted impact of the genetic diversity at locus "AX-89687986" on Y under two water regimes: positive effect of diversity under controlled water deficit conditions (red points and line) and negative effect (ns) under Rain-fed conditions (blue points and line).

Co-localisation

For the GWFA, co-localization with already published QTL were found for almost all the QTLs detected (S3 Table), with a varying number of matches. The 5 SpL F-QTLs co-localised with QTLs associated to the spike characteristics (Unpublished from [61]); [62,63], to plant height [64,65], and yield components traits related to the number of kernels [61] and the weight of kernels [66,67]. The TKW F-QTL co-localised with 5 published QTLs associated to grain quality and weight [68–70] and to heading date [64].

The HE-QTL found for TKW on chr 4A co-localized with QTLs associated to the spike length (unpublished data from [61], plant height [71], grain yield [71], root characteristics [72] and biomass [71] (S4 Table). The HE-QTL found for SpL on the chr 3B co-localized with QTLs associated to the grain yield [71], heading date [73] and root characteristics[72]. The same HE-QTL found for NKM2 and NKS on chr 1B col-localized with grain yield [71], thousand kernels weight [66] and test weight [68].

For the interaction of the significant (F-QTL of the TKW) x Water regime, we found 4 colocalized QTLs in the literature on : the number of kernels/spike, the grain yield per plant, the thousand kernels weight [74] and test weight [64].

S3 Table: QTLs found that their allelic frequency is associated to traits, with their positions on the svevo genome of durum wheat, their –log10 (PValue) and the traits associated to their co-localized QTLs.

*SpL: Spike Length in cm, TKW: Thousand Kernels weight in g, SNP: Single Nucleotide Polymorphism, Peak: physical position of the SNP on the chromosome in base pairs (bp), Lower bound: the minimal physical position that can have the SNP on the chromosome, Upper bound: the maximal physical position that can have the SNP on the chromosome, Chr: chromosome, QTL: Quantitative Trait Loci. The threshold of GWFA –log10(PValue) is 4.46. * Significant test.*

S4 Table: QTLs found that their genetic diversity is associated to traits, with their positions on the svevo genome of durum wheat, their –log 10 (PVal) and the traits associated to their co-localized QTLs.

SpL: Spike Length in cm, NKS: Number of kernels per spike, NKM2: Number of kernels per m^2, TKW: Thousand Kernels weight in g, SNP: Single Nucleotide Polymorphism, Peak: physical position of the SNP on the chromosome in base pairs (bp), Lower bound: the minimal physical position that can have the SNP on the chromosome, Upper bound: the maximal physical position that can have the SNP on the chromosome, Chr: chromosome, QTL: Quantitative Trait Loci.

*The threshold of GWDA –log10(PValue) is 4.46. The threshold of HE vs F test –log10(PValue) is 2.78. * Significant test.*

Global genomic Impact of diversity

The HE slopes on traits were distributed between positive and negative values (e.g, for NKS, Fig. 6). The Wilcoxon tests suggested a significantly larger number of SNPs for which diversity was positively associated with stem length, in both the stress (CWD) and normal (R) treatments (Table 3). This suggests that plants in diverse mixtures tended to grow taller. For yield and its components such as the number of kernels produced (Y, NKM2, NKS), we found a clear excess of SNP whose diversity had a deleterious effect on these traits in the stressful environment, but an excess of SNPs with positive effect or not significant effect in the R treatment. It was the reverse for the grain weight (TKW), where diversity had a positive effect for most SNPs (Table 3). Interestingly, while the spike density per m^2 (NSM2) is one of the most important yield component, no clear global genetic impact was detected.

Fig 6. Slopes distribution of NKS under each environmental condition separately. Blue line for plots under rain-fed conditions. Red line for plots under controlled water deficit conditions.

Table 3. Number of SNPs with a negative and positive effect on traits under CWD and R conditions.

*#: Number, CWD: controlled water deficit conditions, R: rainy conditions, SL: Stem length in cm, SpL: Spike length in cm, NSM2: Number of spikes per m2, NKS: Number of kernels per spike, NKM2: Number of kernels per m2, TKW: Thousand kernel weight in g, Y: Yield in gr/m². Wilcoxon test gives the -log10(PValue) of the SNP pair ranked Wilcoxon test. The significance threshold of the Wilcoxon test is 1.301. * Significant test, ns: non significant test.*

Trait	# of negative slopes in CWD	# of positive slopes in CWD	–log10 (PValue) Wilcoxon Test in CWD	# of negative slopes in in R	# of positive slopes in R	–log10(PValue) Wilcoxon Test in R
SL	5501	7587	>15.65*	5547	7541	>15.65*
SpL	6689	6399	ns	5289	7799	>15.65*
NSM2	6445	6643	ns	6343	6745	ns
NKS	7434	5659	>15.65*	6666	6427	7.05*
NKM2	7349	5739	>15.65*	6742	6346	ns
TKW	5703	7385	>15.65*	5958	7130	>15.65*
Yield	7070	6018	>15.65*	6793	6295	ns

Test of the stress gradient hypothesis

For the traits related to fitness, the stress gradient hypothesis predicts a global positive change in the slopes values under stress compared to the non stressed conditions. The paired Wilcoxon tests was used to explore the changes in the HE slopes from R to CWD at the genome level for the 13K selected SNP (Table 4). The test was significant for most traits except NSM2 and TKW. We found that diversity increased plant height only slightly more under R than under CWD (Table 4). The situation was reversed for yield and its components (NKM2, NKS), where global genetic diversity was more detrimental under stressful conditions than under normal conditions.

Table 4. Difference number and effect of the positive and the negative sum of the difference of slopes in CWD and in R (delta) and the p-value of their wilcoxon's test.

Abbreviation:

*SL : Stem length in cm, SpL : Spike length in cm, NSM2: Number of spikes per m2, NKS: Number of kernels per spike, NKM2: Number of kernels per m2, TKW: Thousand kernel weight in g, Y: Yield in gr/m². $\Delta > 0$, resp., $\Delta < 0$, is the number of SNP for which $\Delta_i = \beta_{CWD,i} - \beta_{R,i}$, is positive, resp. negative, $\beta_{CWD,i}$ and $\beta_{R,i}$ being the slopes of the regression of a trait on the diversity at a given SNPi under control water deficit (CWD) and rainy (R) conditions. Wilcoxon test gives the -log10(PValue) of the SNP pair ranked Wilcoxon test. The significance threshold is 1.301. * Significant test. ns: non significant test.*

Trait	$\Delta > 0$	$\Delta < 0$	Wilcoxon test
SL	6551	6537	6.58*
SpL	5956	7132	>15.65*
NSM2	7042	6046	ns
NKS	6324	6769	13.54*
NKM2	5708	7380	>15.65*
TKW	6510	6578	ns
Yield	5756	7332	>15.65*

Discussion

Summary of the main results and what is new here compared to previous analyses of varietal mixtures.

In the present paper, we investigated the effect of genetic diversity on the performances of a population (here varietal mixtures) under contrasted stress conditions. First, the regression approach was extended to detect the effects of diversity *per se* at any given locus (GWDA). Second, the impact of diversity at a broad genomic scale was also investigated by cumulating results across loci. We detected effects of diversity at four loci (HE-QTL) on several phenotypic traits. Concerning the traits linked with yield and its main components, that can all be considered as proxies of plant fitness, significant HE-QTL were negatively linked to performance and more globally, an excess of loci with a negative impact of diversity *per se* at the genomic scale were identified.

Studies on crop mixtures are still scarce [75–77] and the present experiment is among the few [49] that explored the question using complex mixtures and by explicitly taking into account diversity at the genomic level. In studies or meta-analysis that compare a large number of mixtures with their individual components, a small but positive overyielding is generally

observed. Nevertheless these results also demonstrate the large variability observed between mixtures [27] that goes from clear yield enhancement [76], to significant negative effect [49]. In the present experiment, overyielding was not estimated since our focus was on the factors governing the variation of mixture performances, with the aim to better understand the variation observed for agronomic performance among mixtures and environments.

Our approach is close to other studies using allelic frequencies at many locus on natural populations. These approaches, as the genome-environment association (GEA) analysis often combine a Pool-Seq approach in the aim to uncover SNPs associated to climatic or plant community variation [78–80]. Diversity effects at the genomic level have been reported previously [44,49], but with experimental designs that differed greatly from complete randomness in plant neighbouring, as would be expected under field conditions. Here, we report on a more realistic experiment where both the complexity of the mixtures and the intensity of inter-genotypic plant x plant interactions were high, in order to mimic more closely situations encountered in agronomic conditions.

The challenge of distinguishing the effect of diversity from the effect of allele frequency

Exploring associations between genetic diversity *per se* and phenotypic traits in mixtures, raises a number of methodological challenges and in particular the risk of model overfitting

As any GWAS analysis, the regression approach used is sensitive to false positives due to the large number of tests performed. Using HE as a regressor further increases this risk because the model can overfit the data if there is a direct association between allele frequency and a trait, since it permits to fit non-linear relationships between allele frequencies and the trait. By definition, HE depends on allele frequencies and if two homozygous genotypes have contrasted phenotypic values on average, it is difficult to disentangle the phenotypic effect due to a change in the frequency of the favourable allele from a change of genetic diversity *per se*. For this reason, we were particularly cautious to ascertain our HE-QTLs and we reduced the number of significant associations from 30 to 4, by adding a filter on found HE associations, to be sure that the detected effects were significantly due to the diversity *per se* and not to allelic frequency (tests HE vs. F). It was surprising that the 26 remaining HE-QTL were not detected as F-QTL but this could be due to a non linear association of trait with F or a non-even distribution of allelic frequencies around 0.5, or to a lack of power. By design, the allelic frequencies in each of our 96 mixtures made of 12 genotypes had a reduced between-plot genetic variance compared to classical GWAS performed using individual inbred lines,

hence reducing the statistical power [61]. It also reduced the variation compared to association studies based on pairwise comparisons [49].

Concerning the global genomic and non-parametric approach, we drastically reduced the number of SNPs for which frequencies varied largely among the 96 mixtures to explore correctly the effect of diversity *per se*. As we did not prune the SNP to keep only independent SNP, a level of redundancy shall exist. But as the Wilcoxon tests were highly significant for most traits, including yield, we believe that our results are reasonably solid but deserve to be verified on repeated experiments involving more mixtures in larger sets of environments.

Durum wheat mixture performances are impacted by their genetic composition

Despite the limits detailed above, our results suggest that genetic diversity had a detectable effect on mixtures performances. We are confident about our experimental design and association analyses seen as we found associations between allele frequencies and spike length and TKW that co-localized with QTLs published for the same categories of traits. This proves that the method we used to avoid false positives was efficient and also strengthens our confidence in the results of the GDWA analysis (the four HE-QTLs).

Diversity effect on mixture performance

Table 5 summarizes the main observations on plant height, yield and its components and grain quality. HE-QTL were detected on important traits in each of these categories. The HE-QTLs on the number of kernels per spike (NKS) and on the number of kernels per m^2 (NKM2), two important components of yield, had a negative effect. These traits, along with yield, were also rather negatively correlated with genetic diversity at the global genomic scale (Table 5).

To explain this reduction of the number of kernel per m^2 and yield when diversity increased at least on a part of the genome, one can remark that stem length showed a particular behaviour. While the stress reduced plant height, an interesting trend appeared at the genome global level: SNP whose diversity had a positive impact on stem length were in excess compared to those having a negative impact. This may reflect that plants increased their height as a reaction to the intra genomic complexity of their close environment. Plants increased their carbon allocation into stem and elongation in response to what they could have felt as a more competitive environment. This competition seemed to increase lightly under stress, diversity having a slightly stronger effect on plant elongation in CWD than in R. In our case, this happened despite

the fact that we had restricted the variation in plant height and earliness among the 96 components. In addition, we found that diversity tended to elongate the spikes (SpL), as shown by a positive effect HE-QTL on chr 3B and a genomic excess of SNP whose diversity elongated spikes under R conditions. If spike length is usually expected to reflect the number of kernels per spike, it was not the case here (SpL and NKS were not correlated, Fig. 2). Instead, we postulate that in our mixture condition, the spike elongation may be due to competition (and the positive correlation with stem length) but had no impact on spike fecundity. Instead, this competition-based increased carbon allocation into straw may have resulted in a reduction of the number of kernels per spike (NKS). Indeed, diversity was not favourable for this trait: the HE-QTL detected on the 1B chromosome had a negative impact on NKS in both water regimes and there were also more SNP whose HE had a negative impact than the opposite.

Unlike most components of yield, kernel weight was affected positively by diversity: we found a HE-QTL for TKW on chr 4A with a positive effect and the global effect was also positive (Table 5). These results are very probably linked with the existing trade-off existing between NKM2 and TKW (e.g. [81]). Under this assumption the positive response of TKW to genetic diversity is indirect and driven by the negative answer of NKM2 to the same cue.

Table 5. Synthesis of the impact of diversity on yield and its components
Stress: ↓ significant negative effect of the stress. F and HE-QTL: significant associations with the allelic frequency or the diversity. Global effect: + (resp. -) reports a significant excess of SNP whose diversity had a positive (resp. negative) effect on the trait. Stress gradient hypothesis: "Yes" if a significantly more SNP had a more positive impact of their diversity under CWD than under R, "No" in the reverse case, ns: non-significant..

R: Rainy conditions, CWD: controlled water deficit conditions, SL: stem length, SpL: spike lenght, NSM2: number of spikes per m², NKS: number of kernels per spike, NKM2: number of kernels per m², TKW: Thousand-kernel weight in g, Y: yield in g/m².

	Stress	F-QTL	HE-QTL	Global effect		Stress gradient hypothesis
				R	CWD	
SL	↓			+	+	Yes
SpL		1B 2B 4B 5A	3B (+)	+		No
NSM2	↓					ns
NKS	↓		1B (-)	-	-	No
NKM2	↓		1B (-)		-	No

			2A GxE			
TKW	↓	2A<br>7B GxE	4A (+)	+	+	ns
Y	↓		2A GxE		-	No

Ecological and evolutionary mechanisms involved

Overall, our results disagree with the prediction that niche complementarity and facilitation should increase productivity when genetic diversity increases [23,82]. A negative effect of genetic diversity on the yield of neighbouring lines of durum wheat was found previously [46], even if a global overyielding effect was observed on average[49]. This negative effect of diversity might be explained in the framework of the kin selection theory with the green beard effect [32,44,45]. Green-beard alleles are expected to promote altruism toward individuals who share a gene that is expressed by a specific phenotypic trait. If applied to crop mixtures, this effect would therefore predict that mixtures in which genotypes share the same allele at some locus are more productive than mixtures in which genotypes have different alleles [83]. Two hypotheses of the negative effect of allelic diversity on yield, based on the green beard theory can be proposed: i) a negative plant x plant interaction between individuals carrying different alleles at the same locus, such as a competitive effect, ii) a positive interaction between individuals carrying identical alleles at the locus such a cooperative effect [84]. So in durum wheat, diversity at some locus might oppose to a global positive effect of varietal diversity at other loci. Unfortunately here, yield data were not available for the 96 pure components and we could not test if we had a global beneficial overyielding impact in mixtures, as found by [49].

The He-QTL on the chr 1B with a negative effect on the number of kernels per spike (NKS and Y) co-localizes with two published QTLs, one associated to grain yield [71] and another to test weight [68]. The QTL boundaries is of 36,229 bp in which we found a gene "TRITD1Bv1G013230" located in the [32,914,033 - 32,915,768] boundaries, and found responsible of the TOX high mobility group box protein, putative (DUF1635). In the literature, this gene has not a function identified in plants. This HE-QTL would be a particularly interesting candidate for a so-called green beard locus, whose diversity is therefore expected to reduce the performance of the group. It is possible that the HE-QTL on chr 1B includes a gene involved in one of the biochemical pathways implied in plant communication and recognition, such as the allelochemical DIMBOA, strigolactone or COV [85–87], but we have no candidate yet. It may

be first interesting to confirm this HE-QTL by experimenting in mixtures with a simplified composition, to reduce the confidence interval of the QTL and to annotate more precisely the genome area.

Even if the interpretation of the mechanisms involved remains speculative, the effects of diversity detected here are consistent with previous observations showing that, beyond the average phenotypic value predicted from the values of pure components, plant x plant interactions are also at play and may explain part of the among mixture variation.

Mixtures, diversity and stress

One of the factors that could complexity plant x plant interactions in mixtures, and explain the observed high variability in the effects of diversity, is environmental conditions. The stress gradient hypothesis [34] applied to agricultural conditions predicts that a stressful environment should increase the positive effects of diversity in mixtures compared to the standard conditions experienced by plants in intensive agriculture. Previous studies provided support for the stress gradient hypothesis in the context of crop mixtures, by showing that over-yielding increased under high disease pressure [27] or that mixtures were more tolerant to environmental stresses [88]. Among the mechanisms that might be responsible for these effects, stresses could foster positive interactions, complementarity and compensation between the varieties of the mixtures.

Stress induced by the water deficit

In our experiment, the controlled water deficit induced a measurable stress compared to the rainy conditions. Under water stress we observed (smaller and earlier-flowering plants), effects that are consistent with previous results [89–91]. Plasticity leading to early flowering time and a shorter vegetative phase is seen as an important and classical adaptive feature for wheat production under drought to minimize exposure to dehydration during the sensitive flowering and post-anthesis grain filling periods and to complete their life-cycle quickly during the brief period of favourable conditions [91,92]. Water stress also affected the reproductive success of plants, diminishing slightly the number of spike per m². The water stress imposed at the end of tillering, when the number of spikes is determined [93,94] was mild, but increased during the rest of the crop cycle, in particular during the stem elongation or jointing [90]. This may have resulted in a stronger regression of tillers, causing a final decrease of NSM2 under water stress. The stress then impacted the number of kernels per

spike (NKS) during spike growth [94]. Yet, drought also depleted the kernel weight in spite of the lower competitin for assimilates between kernels induced by the lower NKM2. Overall, this lead to a strong reduction in yield (Y), as commonly reported [95–97].

Test of the stress gradient hypothesis

For two HE-QTLs on yield (as well as NKM2), we found an interaction with the environment, and the slope was positive under stress conditions only. These results are in agreement with the stress gradient hypothesis that predicts mixtures could benefit from their genetic diversity at some loci when growing in non-optimal abiotic conditions. Under stress, the diversity at these loci may reflect a phenotypic complementarity between the components within mixtures, leading to an improvement in the access to resources , water in particular [98–100]. A compensation mechanism may also explain the stress gradient hypothesis if some varieties are affected by the stress during a critical development phase while others escape it and eventually compensate partly the yield [101,102].

At the global genetic level, however, we found that global genetic diversity was more detrimental under stressful conditions than under normal conditions for fitness related traits such as NKS, NKM2 and yield. If positive effects were observed on a number of loci, the distribution was overall skewed towards negative impacts of diversity under stress (e.g. Fig. 5). This is not in favour of the stress gradient hypothesis. Plant height was the only trait that validated the stress gradient hypothesis as the global genetic level, but as explained in the previous paragraph, this might be an effect of the perceived level of competition.

Overall, our experiment provides limited support for the stress gradient hypothesis, thereby suggesting that complementarity or compensation do not mitigate the effects of stress in our mixtures. Instead, competition is harsher in the stressful environment and negatively affects the yield. In their meta-analysis, Borg et al. (2018) were also unsuccessful in their attempt to test the influence of abiotic stress, due to a lack of information (not enough studies and not enough details about the nature of the stress) [27]. This underlines the need for further studies to better understand the ecological mechanisms that determine mixtures performances, in particular the relative roles of competition versus complementarity.

Guidelines to assemble genotypes in a mixture

Our results highlight complex and contradictory mechanisms at play in our 96 mixtures. Finding variable effects in our experiment mirrors large variability in the over-yielding reported among mixtures in the literature. Finding significant associations between yield or its components and

the diversity at specific loci is a methodological proof of concept that plant x plant interactions can shape the performance of crop mixtures. Even if there is an excess of negative associations between diversity and yield, a large number of loci still have a positive effect (Fig 5). Ultimately, assembling mixtures by selecting genotypes that differ on some loci for which there is evidence of complementarity or compensation, but remain uniform for others loci, such as candidate green-beard locus. This blind approach cold complement trait based assembly rules [103].

Funding information:

The EU H2020 SOLACE funded the experiment; PA received a grant from l'Assocation de Spécialisation et d'Orientation Scientifique du Liban.

References:

1. Brisson N, Gate P, Gouache D, Charmet G, Oury F-X, Huard F. Why are wheat yields stagnating in Europe? A comprehensive data analysis for France. Field Crops Res. 2010;119: 201–212. doi:10.1016/j.fcr.2010.07.012

2. Chapman SC, Chakraborty S, Dreccer MF, Howden SM. Plant adaptation to climate change—opportunities and priorities in breeding. Crop Pasture Sci. 2012;63: 251–268. doi:10.1071/CP11303

3. Loreau M. Biodiversity and ecosystem functioning: recent theoretical advances. Oikos. 2000;91: 3–17.

4. Oury F-X, Godin C, Mailliard A, Chassin A, Gardet O, Giraud A, et al. A study of genetic progress due to selection reveals a negative effect of climate change on bread wheat yield in France. Eur J Agron. 2012;40: 28–38. doi:10.1016/j.eja.2012.02.007

5. Tilman D, Cassman KG, Matson PA, Naylor R, Polasky S. Agricultural sustainability and intensive production practices. Nature. 2002;418: 671–677. doi:10.1038/nature01014

6. Ben-Ari T, Boé J, Ciais P, Lecerf R, Velde MV der, Makowski D. Causes and implications of the unforeseen 2016 extreme yield loss in the breadbasket of France. Nat Commun. 2018;9: 1–10. doi:10.1038/s41467-018-04087-x

7. van Ittersum MK, Cassman KG, Grassini P, Wolf J, Tittonell P, Hochman Z. Yield gap analysis with local to global relevance—A review. Field Crops Res. 2013;143: 4–17. doi:10.1016/j.fcr.2012.09.009

8. Goldringer I, Bonnin I, David J. Rôle de la sélection dans la dynamique de la diversité des Blés cultivés : de la domestication à la sélection moderne au 20ème siècle. Innov Agron. 2017;29: 1–15.

9. Østergård H, Finckh MR, Fontaine L, Goldringer I, Hoad SP, Kristensen K, et al. Time for a shift in crop production: embracing complexity through diversity at all levels. J Sci Food Agric. 2009;89: 1439–1445. doi:10.1002/jsfa.3615

10. FAO. The Second Report on the State of the World's Plant Genetic Resources for Food and Agriculture. 2010 [cited 27 Apr 2021]. Available: http://www.fao.org/3/i1500e/i1500e00.htm

11. Beillouin D, Ben-Ari T, Malezieux E, Seufert V, Makowski D. Positive but variable effects of crop diversification on biodiversity and ecosystem services. Glob Change Biol. 2021.

12. Kiær L, Skovgaard I, Østergård H. Grain yield increase in cereal variety mixtures: A meta-analysis of field trials. Field Crops Res. 2009;114: 361–373. doi:10.1016/j.fcr.2009.09.006

13. Mundt CC. Use of Multiline Cultivars and Cultivar Mixtures for Disease Management. Annu Rev Phytopathol. 2002;40: 381–410. doi:10.1146/annurev.phyto.40.011402.113723

14. Altieri MA. The ecological role of biodiversity in agroecosystems. Invertebrate biodiversity as bioindicators of sustainable landscapes. Elsevier; 1999. pp. 19–31.

15. Isbell F. Agroecology: agroecosystem diversification. Nat Plants. 2015;1: 1–2.

16. Malézieux E. Designing cropping systems from nature. Agron Sustain Dev. 2012;32: 15–29.

17. Smithson JB, Lenné JM. Varietal mixtures: a viable strategy for sustainable productivity in subsistence agriculture. Ann Appl Biol. 1996;128: 127–158. doi:10.1111/j.1744-7348.1996.tb07096.x

18. Brousseau L, Bonal D, Cigna J, Scotti I. Highly local environmental variability promotes intrapopulation divergence of quantitative traits: an example from tropical rain forest trees. Ann Bot. 2013;112: 1169–1179. doi:10.1093/aob/mct176

19. Hughes AR, Inouye BD, Johnson MTJ, Underwood N, Vellend M. Ecological consequences of genetic diversity. Ecol Lett. 2008;11: 609–623. doi:https://doi.org/10.1111/j.1461-0248.2008.01179.x

20. Creissen HE, Jorgensen TH, Brown JK. Increased yield stability of field-grown winter barley (Hordeum vulgare L.) varietal mixtures through ecological processes. Crop Prot. 2016;85: 1–8.

21. Lannou C, Mundt CC. Evolution of a pathogen population in host mixtures: rate of emergence of complex races. Theor Appl Genet. 1997;94: 991–999. doi:10.1007/s001220050506

22. Wolfe MS. The Current Status and Prospects of Multiline Cultivars and Variety Mixtures for Disease Resistance. Annu Rev Phytopathol. 1985;23: 251–273. doi:10.1146/annurev.py.23.090185.001343

23. Zhu Y, Chen H, Fan J, Wang Y, Li Y, Chen J, et al. Genetic diversity and disease control in rice. Nature. 2000;406: 718–722. doi:10.1038/35021046

24. Enjalbert J, Litrico I, Fournier E, Médiène S, Gauffreteau A, Borg J, et al. Mélanges variétaux et mélanges plurispécifiques – atouts et contraintes. Innov Agron. 2019;75: 49–71.

25. Vidal T, Saint- Jean S, Lusley P, Leconte M, Ben Krima S, Boixel A, et al. Cultivar mixture effects on disease and yield remain despite diversity in wheat height and earliness. Plant Pathol. 2020;69: 1148–1160. doi:10.1111/ppa.13200

26. Weigelt A, Jolliffe P. Indices of Plant Competition. J Ecol. 2003;91: 707–720.

27. Borg J, Kiær LP, Lecarpentier C, Goldringer I, Gauffreteau A, Saint-Jean S, et al. Unfolding the potential of wheat cultivar mixtures: A meta-analysis perspective and identification of knowledge gaps. Field Crops Res. 2018;221: 298–313. doi:10.1016/j.fcr.2017.09.006

28. Reiss ER, Drinkwater LE. Cultivar mixtures: a meta-analysis of the effect of intraspecific diversity on crop yield. Ecol Appl. 2018;28: 62–77. doi:10.1002/eap.1629

29. Macarthur R, Levins R. The Limiting Similarity, Convergence, and Divergence of Coexisting Species. Am Nat. 1967;101: 377–385. doi:10.1086/282505

30. Stachowicz JJ. Mutualism, Facilitation, and the Structure of Ecological Communities. BioScience. 2001;51: 235. doi:10.1641/0006-3568(2001)051[0235:MFATSO]2.0.CO;2

31. Cardinale BJ, Palmer MA, Collins SL. Species diversity enhances ecosystem functioning through interspecific facilitation. Nature. 2002;415: 426–429. doi:10.1038/415426a

32. Hamilton WD. The genetical evolution of social behaviour. I. J Theor Biol. 1964;7: 1–16. doi:10.1016/0022-5193(64)90038-4

33. Murphy GP, Swanton CJ, Acker RCV, Dudley SA. Kin recognition, multilevel selection and altruism in crop sustainability. J Ecol. 2017;105: 930–934. doi:10.1111/1365-2745.12787

34. Bertness MD, Callaway R. Positive interactions in communities. Trends Ecol Evol. 1994;9: 191–193. doi:10.1016/0169-5347(94)90088-4

35. Brooker RW, Maestre FT, Callaway RM, Lortie CL, Cavieres LA, Kunstler G, et al. Facilitation in plant communities: the past, the present, and the future. J Ecol. 2008;96: 18–34. doi:10.1111/j.1365-2745.2007.01295.x

36. Bruno JF, Stachowicz JJ, Bertness MD. Inclusion of facilitation into ecological theory. Trends Ecol Evol. 2003;18: 119–125. doi:10.1016/S0169-5347(02)00045-9

37. Callaway RM, Walker LR. Competition and Facilitation: A Synthetic Approach to Interactions in Plant Communities. Ecology. 1997;78: 1958–1965. doi:10.2307/2265936

38. Holmgren M, Scheffer M, Huston MA. The Interplay of Facilitation and Competition in Plant Communities. Ecology. 1997;78: 1966–1975. doi:10.1890/0012-9658(1997)078[1966:TIOFAC]2.0.CO;2

39. Yachi S, Loreau M. Biodiversity and ecosystem productivity in a fluctuating environment: the insurance hypothesis. Proc Natl Acad Sci. 1999;96: 1463–1468.

40. Barot S, Allard V, Cantarel A, Enjalbert J, Gauffreteau A, Goldringer I, et al. Designing mixtures of varieties for multifunctional agriculture with the help of ecology. A review. Agron Sustain Dev. 2017;37: 13. doi:10.1007/s13593-017-0418-x

41. Newton A. Plant Disease Control through the use of Variety Mixtures. 2009. pp. 162–171. doi:10.1002/9781444312157.ch8

42. Metzker ML. Sequencing technologies - the next generation. Nat Rev Genet. 2010;11: 31–46. doi:10.1038/nrg2626

43. Rimbert H, Darrier B, Navarro J, Kitt J, Choulet F, Leveugle M, et al. High throughput SNP discovery and genotyping in hexaploid wheat. PloS One. 2018;13: e0186329.

44. Wuest SE, Niklaus PA. A plant biodiversity effect resolved to a single chromosomal region. Nat Ecol Evol. 2018;2: 1933–1939.

45. Turner KG, Lorts CM, Haile AT, Lasky JR. Effects of genomic and functional diversity on stand-level productivity and performance of non-native Arabidopsis. Proc R Soc B. 2020;287: 20202041.

46. Montazeaud G, Flutre T, Ballini E, Morel J-B, David J, Girodolle J, et al. From cultivar mixtures to allelic mixtures: opposite effects of allelic richness between genotypes and genotype richness in wheat. in revision.

47. Yang Y, Tilman D, Furey G, Lehman C. Soil carbon sequestration accelerated by restoration of grassland biodiversity. Nat Commun. 2019;10: 1–7.

48. Nachit M, Picard E, Monneveux P, Labhilili M, Baum M, Rivoal R. Présentation d'un programme international d'amélioration du blé dur pour le bassin méditerranéen. Cah Agric. 1998;7: 510-515 (1).

49. Montazeaud G, Violle C, Roumet P, Rocher A, Ecarnot M, Compan F, et al. Multifaceted functional diversity for multifaceted crop yield: Towards ecological assembly rules for varietal mixtures. J Appl Ecol. 2020;57: 2285–2295. doi:10.1111/1365-2664.13735

50. David J, Holtz Y, Ranwez V, Santoni S, Sarah G, Ardisson M, et al. Genotyping by sequencing transcriptomes in an evolutionary pre-breeding durum wheat population. Mol Breed. 2014;34: 1531–1548. doi:10.1007/s11032-014-0179-z

51. Ballini E, Tavaud M, Ducasse A, Sanchez D, Paux E, Kitt J, et al. Genome wide association mapping for resistance to multiple fungal pathogens in a panel issued from a broad composite cross-population of tetraploid wheat Triticum turgidum. Euphytica. 2020;216: 92. doi:10.1007/s10681-020-02631-9

52. Donald CM. The breeding of crop ideotypes. Euphytica. 1968;17: 385–403. doi:10.1007/BF00056241

53. Ford ED, Diggle PJ. Competition for Light in a Plant Monoculture Modelled as a Spatial Stochastic Process. Ann Bot. 1981;48: 481–500. doi:10.1093/oxfordjournals.aob.a086152

54. Maccaferri M, Harris NS, Twardziok SO, Pasam RK, Gundlach H, Spannagl M, et al. Durum wheat genome highlights past domestication signatures and future improvement targets. Nat Genet. 2019;51: 885–895. doi:10.1038/s41588-019-0381-3

55. Nei M. Estimation of average heterozygosity and genetic distance from a small number of individuals. Genetics. 1978;89: 583–590.

56. Plackett RL. Karl Pearson and the chi-squared test. Int Stat Rev Int Stat. 1983; 59–72.

57. Galwey NW. A new measure of the effective number of tests, a practical tool for comparing families of non-independent significance tests. Genet Epidemiol. 2009;33: 559–568. doi:10.1002/gepi.20408

58. Cormier F, Le Gouis J, Dubreuil P, Lafarge S, Praud S. A genome-wide identification of chromosomal regions determining nitrogen use efficiency components in wheat (Triticum aestivum L.). Theor Appl Genet. 2014;127: 2679–2693. doi:10.1007/s00122-014-2407-7

59. Hill WG, Robertson A. Linkage disequilibrium in finite populations. Theor Appl Genet. 1968;38: 226–231.

60. Breseghello F, Sorrells ME. Association Mapping of Kernel Size and Milling Quality in Wheat (Triticum aestivum L.) Cultivars. Genetics. 2006;172: 1165–1177. doi:10.1534/genetics.105.044586

61. Soriano JM, Colasuonno P, Marcotuli I, Gadaleta A. Meta-QTL analysis and identification of candidate genes for quality, abiotic and biotic stress in durum wheat. Sci Rep. 2021;11: 11877. doi:10.1038/s41598-021-91446-2

62. Faris JD, Zhang Q, Chao S, Zhang Z, Xu SS. Analysis of agronomic and domestication traits in a durum × cultivated emmer wheat population using a high-density single nucleotide polymorphism-based linkage map. Theor Appl Genet. 2014;127: 2333–2348. doi:10.1007/s00122-014-2380-1

63. Soriano JM, Malosetti M, Roselló M, Sorrells ME, Royo C. Dissecting the old Mediterranean durum wheat genetic architecture for phenology, biomass and yield formation by association mapping and QTL meta-analysis. PLOS ONE. 2017;12: e0178290. doi:10.1371/journal.pone.0178290

64. Maccaferri M, Sanguineti MC, Demontis A, El-Ahmed A, Garcia del Moral L, Maalouf F, et al. Association mapping in durum wheat grown across a broad range of water regimes. J Exp Bot. 2011;62: 409–438. doi:10.1093/jxb/erq287

65. Maccaferri M, Sanguineti MC, Corneti S, Ortega JLA, Salem MB, Bort J, et al. Quantitative Trait Loci for Grain Yield and Adaptation of Durum Wheat (Triticum durum Desf.) Across a Wide Range of Water Availability. Genetics. 2008;178: 489–511. doi:10.1534/genetics.107.077297

66. Peleg Z, Fahima T, Korol AB, Abbo S, Saranga Y. Genetic analysis of wheat domestication and evolution under domestication. J Exp Bot. 2011;62: 5051–5061. doi:10.1093/jxb/err206

67. Peng J, Ronin Y, Fahima T, Röder MS, Li Y, Nevo E, et al. Domestication quantitative trait loci in Triticum dicoccoides, the progenitor of wheat. Proc Natl Acad Sci. 2003;100: 2489–2494. doi:10.1073/pnas.252763199

68. Canè MA, Maccaferri M, Nazemi G, Salvi S, Francia R, Colalongo C, et al. Association mapping for root architectural traits in durum wheat seedlings as related to agronomic performance. Mol Breed New Strateg Plant Improv. 2014;34: 1629–1645. doi:10.1007/s11032-014-0177-1

69. Colasuonno P, Lozito ML, Marcotuli I, Nigro D, Giancaspro A, Mangini G, et al. The carotenoid biosynthetic and catabolic genes in wheat and their association with yellow pigments. BMC Genomics. 2017;18: 122. doi:10.1186/s12864-016-3395-6

70. Fiedler JD, Salsman E, Liu Y, Michalak de Jiménez M, Hegstad JB, Chen B, et al. Genome-Wide Association and Prediction of Grain and Semolina Quality Traits in Durum Wheat Breeding Populations. Plant Genome. 2017;10: plantgenome2017.05.0038. doi:10.3835/plantgenome2017.05.0038

71. Mengistu DK, Kidane YG, Catellani M, Frascaroli E, Fadda C, Pè ME, et al. High-density molecular characterization and association mapping in Ethiopian durum wheat landraces reveals high diversity and potential for wheat breeding. Plant Biotechnol J. 2016;14: 1800–1812. doi:10.1111/pbi.12538

72. Maccaferri M, El-Feki W, Nazemi G, Salvi S, Canè MA, Colalongo MC, et al. Prioritizing quantitative trait loci for root system architecture in tetraploid wheat. J Exp Bot. 2016;67: 1161–1178.

73. Maccaferri M, Cane' MA, Sanguineti MC, Salvi S, Colalongo MC, Massi A, et al. A consensus framework map of durum wheat (Triticum durum Desf.) suitable for linkage disequilibrium analysis and genome-wide association mapping. BMC Genomics. 2014;15: 873. doi:10.1186/1471-2164-15-873

74. Mangini G, Gadaleta A, Colasuonno P, Marcotuli I, Signorile AM, Simeone R, et al. Genetic dissection of the relationships between grain yield components by genome-wide association mapping in a collection of tetraploid wheats. Zhang A, editor. PLOS ONE. 2018;13: e0190162. doi:10.1371/journal.pone.0190162

75. Borg J, Kiær LP, Lecarpentier C, Goldringer I, Gauffreteau A, Saint-Jean S, et al. Unfolding the potential of wheat cultivar mixtures: A meta-analysis perspective and identification of knowledge gaps. Field Crops Res. 2018;221: 298–313.

76. Elhakeem A, Bastiaans L, Houben S, Couwenberg T, Makowski D, van der Werf W. Do cover crop mixtures give higher and more stable yields than pure stands? Field Crops Res. 2021;270: 108217. doi:10.1016/j.fcr.2021.108217

77. Smith RG, Warren ND, Cordeau S. Are cover crop mixtures better at suppressing weeds than cover crop monocultures? Weed Sci. 2020;68: 186–194. doi:10.1017/wsc.2020.12

78. Frachon L, Bartoli C, Carrère S, Bouchez O, Chaubet A, Gautier M, et al. A Genomic Map of Climate Adaptation in Arabidopsis thaliana at a Micro-Geographic Scale. Front Plant Sci. 2018;9: 967. doi:10.3389/fpls.2018.00967

79. Rellstab C, Zoller S, Tedder A, Gugerli F, Fischer M. Validation of SNP Allele Frequencies Determined by Pooled Next-Generation Sequencing in Natural Populations of a Non-Model Plant Species. PloS One. 2013;8: e80422. doi:10.1371/journal.pone.0080422

80. Santos AS, Gaiotto FA. Knowledge status and sampling strategies to maximize cost-benefit ratio of studies in landscape genomics of wild plants. Sci Rep. 2020;10: 1–9.

81. Slafer GA, Elia M, Savin R, García GA, Terrile II, Ferrante A, et al. Fruiting efficiency: an alternative trait to further rise wheat yield. Food Energy Secur. 2015;4: 92–109. doi:10.1002/fes3.59

82. Finckh M, Gacek E, Goyeau H, Lannou C, Merz U, Mundt C, et al. Cereal variety and species mixtures in practice, with emphasis on disease resistance. Agronomie. 2000;20: 813–837.

83. Fréville H, Roumet P, Rode NO, Rocher A, Latreille M, Muller M-H, et al. Preferential helping to relatives: A potential mechanism responsible for lower yield of crop variety mixtures? Evol Appl. 2019;12: 1837–1849.

84. Gardner A. The greenbeard effect. Curr Biol. 2019;29: R430–R431. doi:10.1016/j.cub.2019.03.063

85. Hazrati H, Fomsgaard IS, Kudsk P. Root-Exuded Benzoxazinoids: Uptake and Translocation in Neighboring Plants. J Agric Food Chem. 2020;68: 10609–10617. doi:10.1021/acs.jafc.0c04245

86. Li Y-H, Xia Z-C, Kong C-H. Allelobiosis in the interference of allelopathic wheat with weeds. Pest Manag Sci. 2016;72: 2146–2153. doi:10.1002/ps.4246

87. Zhang S-Z, Li Y-H, Kong C-H, Xu X-H. Interference of allelopathic wheat with different weeds. Pest Manag Sci. 2016;72: 172–178. doi:10.1002/ps.3985

88. Creissen HE, Jorgensen TH, Brown JKM. Stabilization of yield in plant genotype mixtures through compensation rather than complementation. Ann Bot. 2013;112: 1439–1447. doi:10.1093/aob/mct209

89. Flohr B, Hunt JR, Kirkegaard J, Evans J. Water and temperature stress define the optimal flowering period for wheat in south-eastern Australia. Field Crops Res. 2017;209: 108–119. doi:10.1016/j.fcr.2017.04.012

90. Gate P. Ecophysiologie du Blé: de la plante à la culture. Paris : Technique et Documentation-Lavoisier. 1995. Available: https://infodoc.agroparistech.fr/index.php?lvl=notice_display&id=34311

91. Shavrukov Y, Kurishbayev A, Jatayev S, Shvidchenko V, Zotova L, Koekemoer F, et al. Early Flowering as a Drought Escape Mechanism in Plants: How Can It Aid Wheat Production? Front Plant Sci. 2017;8: 1950. doi:10.3389/fpls.2017.01950

92. Turner NC. Adaptation to water deficits: a changing perspective. Funct Plant Biol. 1986;13: 175–190.

93. Gate P, Grimaud L. L'épi de blé à la floraison : un outil remarquable pour l'analyse et la prévision du nombre de grains. 1989.

94. Masle-Meynard J, Sebillotte M. Etude de l'hétérogénéité d'un peuplement de blé d'hiver. II. - Origine des différentes catégories d'individus du peuplement ; éléments de description de sa structure. Agronomie. 1981;1: 217–224.

95. Ashraf MY, Naqvi SSM. Studies on water uptake, germination and seedling growth of wheat genotypes under PEG-600 induced water stress. Pak J Sci Ind Res. 1995;38: 130–133.

96. Akram M. Growth And Yield Components Of Wheat Under Water Stress Of Different Growth Stages. Bangladesh J Agric Res. 2011;36: 455–468. doi:10.3329/bjar.v36i3.9264

97. Pour-Aboughadareh A, Mohammadi R, Etminan A, Shooshtari L, Maleki-Tabrizi N, Poczai P. Effects of Drought Stress on Some Agronomic and Morpho-Physiological Traits in Durum Wheat Genotypes. Sustainability. 2020;12: 5610. doi:10.3390/su12145610

98. Essah SYC, Stoskopf NC. Mixture performance of phenotypically contrasting barley cultivars. Can J Plant Sci. 2002;82: 1–6. doi:10.4141/P01-043

99. Francis CA. Biological efficiencies in multiple-cropping systems. Adv Agron. 1989;42: 1–42.

100. Sarandon SJ, Sarandon R. Mixture of cultivars: pilot field trial of an ecological alternative to improve production or quality of wheat (Triticum aestivum). J Appl Ecol. 1995; 288–294.

101. Sammons DJ, Baenziger PS. Performance of four winter wheat cultivars in blended populations. Field Crops Res. 1985;10: 135–142. doi:10.1016/0378-4290(85)90021-8

102. Stützel H, Aufhammer W. The physiological causes of mixing effects in cultivar mixtures: A general hypothesis. Agric Syst. 1990;32: 41–53.

103. Litrico I, Violle C. Diversity in plant breeding: a new conceptual framework. Trends Plant Sci. 2015;20: 604–612. doi:10.1016/j.tplants.2015.07.007

Supporting information captions:

S1 Fig. The distribution of plant height of the 96 EPOs selected from the 180 lines of EPOs extracted for our experimentation. Red bars: 180 lines extracted from EPOs. Blue bars: 96 selected EPOs for the experiment, with uniform plant height.

S2 Fig. The distribution of earliness of the 96 EPOs selected from the 180 lines of EPOs extracted for our experimentation. Red bars: 180 lines extracted from EPOs. Blue bars: 96 selected EPOs for the experiment, with uniform plant height.

S3 Fig. Genome wide Association between allelic frequencies and TKW on a set of 96 12 way mixture and 96,582 bi allelic SNP. (A) Manhattan plot of the GWFA of TKW and 96k SNPs distributed along the durum wheat genome. The solid blue line represents the Family-Wise Error Rate (FWER) of 5% computed with the Galwey method (with a value of 4.56). (B) Relation between the allelic frequency of the peak SNP “AX-89444977” and TKW. Red points corresponds to plots under controlled water deficit conditions. Black points corresponds to plots under rainy conditions.

S1 Table. SNPs having significant associations between their allelic frequencies and the yield and others traits, with their –log10(PValue) and their positions on the chromosomes.

SpL: Spike Length in cm, TKW: Thousand Kernels weight in g, SNP: Single Nucleotide Polymorphism, Peak: physical position of the SNP on the chromosome in base pairs (bp), Lower bound: the minimal physical position that can have the SNP on the chromosome, Upper bound: the maximal physical position that can have the SNP on the chromosome, Chr: chromosome, QTL: Quantitative Trait Loci.

*The threshold of GWFA –log10(PValue) is 4.46. When GWFA –log10(PValue) is > 4.46 the association between the trait and the QTL is significant, and vice versa. * Significant –log10(PValue).*

S2 Table: QTLs having significant associations between their genetic diversity, the yield, and its components with the sign of the effect of their genetic diversity on each trait and their positions on chromosomes.

SL: Stem length in cm, SpL: Spike Length in cm, NKS: Number of kernels per spike, NKM2: Number of kernels per m^2*, TKW: Thousand Kernels weight in g, Y: yield in gr/*m^2*, SNP: Single Nucleotide Polymorphism, Peak: physical position of the SNP on the chromosome in base pairs (bp), Lower bound: the minimal physical position that can have the SNP on the chromosome, Upper bound: the maximal physical position that can have the SNP on the chromosome, Chr: chromosome, QTL: Quantitative Trait Loci.*

*The threshold of GWDA –log10(PValue) is 4.46. When GWDA –log10(PValue) is > 4.46 the association between the trait and the QTL is significant, and vice versa. * Significant –log10(PValue).*

S3 Table: QTLs found that their allelic frequency is associated to traits, with their positions on the svevo genome of durum wheat, their –log10 (PValue) and the traits associated to their co-localized QTLs.

SpL: Spike Length in cm, TKW: Thousand Kernels weight in g, SNP: Single Nucleotide Polymorphism, Peak: physical position of the SNP on the chromosome in base pairs (bp), Lower bound: the minimal physical position

that can have the SNP on the chromosome, Upper bound: the maximal physical position that can have the SNP on the chromosome, Chr: chromosome, QTL: Quantitative Trait Loci.

The threshold of GWFA –log10(PValue) is 4.46. When GWFA –log10(PValue) is > 4.46 the association between the trait and the QTL is significant, and vice versa. * Significant –log10(PValue).

S4 Table: The four QTLs left that their genetic diversity is associated to traits, with their positions on the svevo genome of durum wheat, their –log 10 (PVal) and the traits associated to their co-localized QTLs.

SpL: Spike Length in cm, NKS: Number of kernels per spike, NKM2: Number of kernels per m^2, TKW: Thousand Kernels weight in g, SNP: Single Nucleotide Polymorphism, Peak: physical position of the SNP on the chromosome in base pairs (bp), Lower bound: the minimal physical position that can have the SNP on the chromosome, Upper bound: the maximal physical position that can have the SNP on the chromosome, Chr: chromosome, QTL: Quantitative Trait Loci.

The threshold of GWDA –log10(PValue) is 4.46. When GWDA –log10(PValue) is > 4.46 the association between the trait and the QTL is significant, and vice versa. The threshold of HE vs F test –log10(PValue) is 2.78. When HE vs F test –log10(PValue) is > 2.78 the effect of the QTL is considered as an effect of genetic diversity. * Significant –log10(PValue).

Click here to access/download;Figure;Fig 1.tif

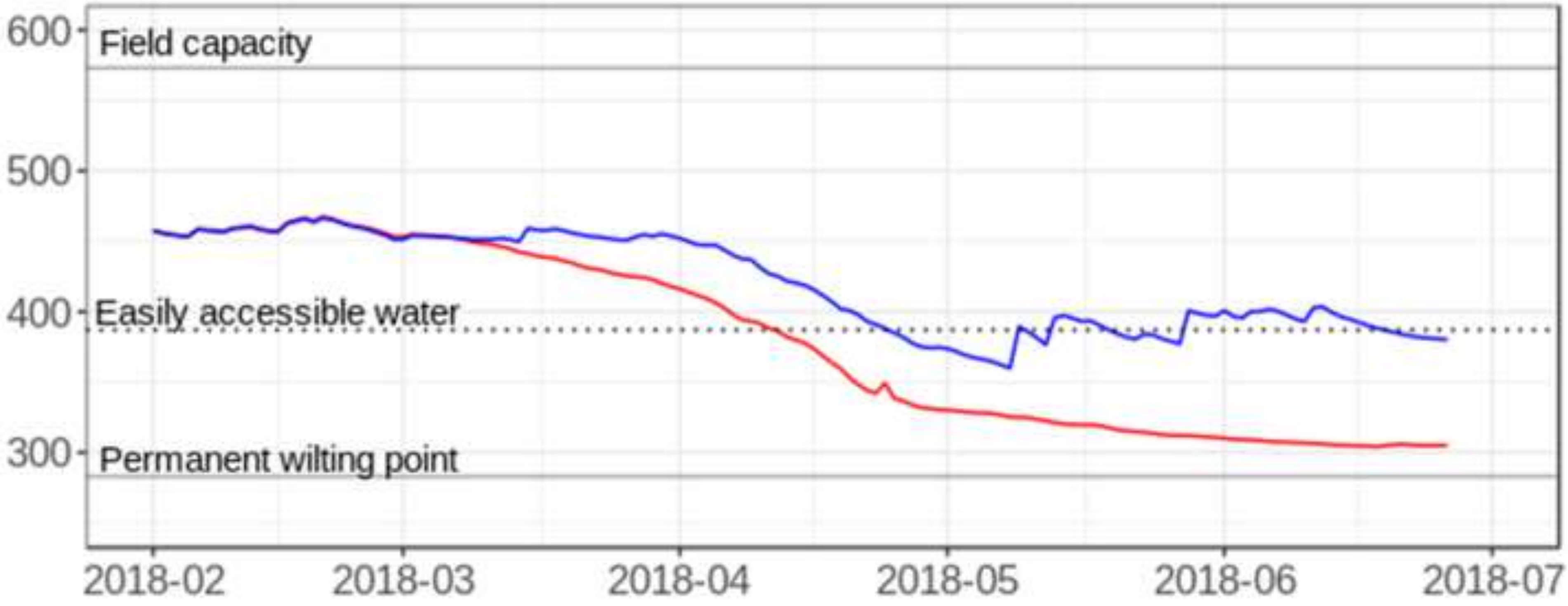

Click here to access/download;Figure;Fig 2.tif

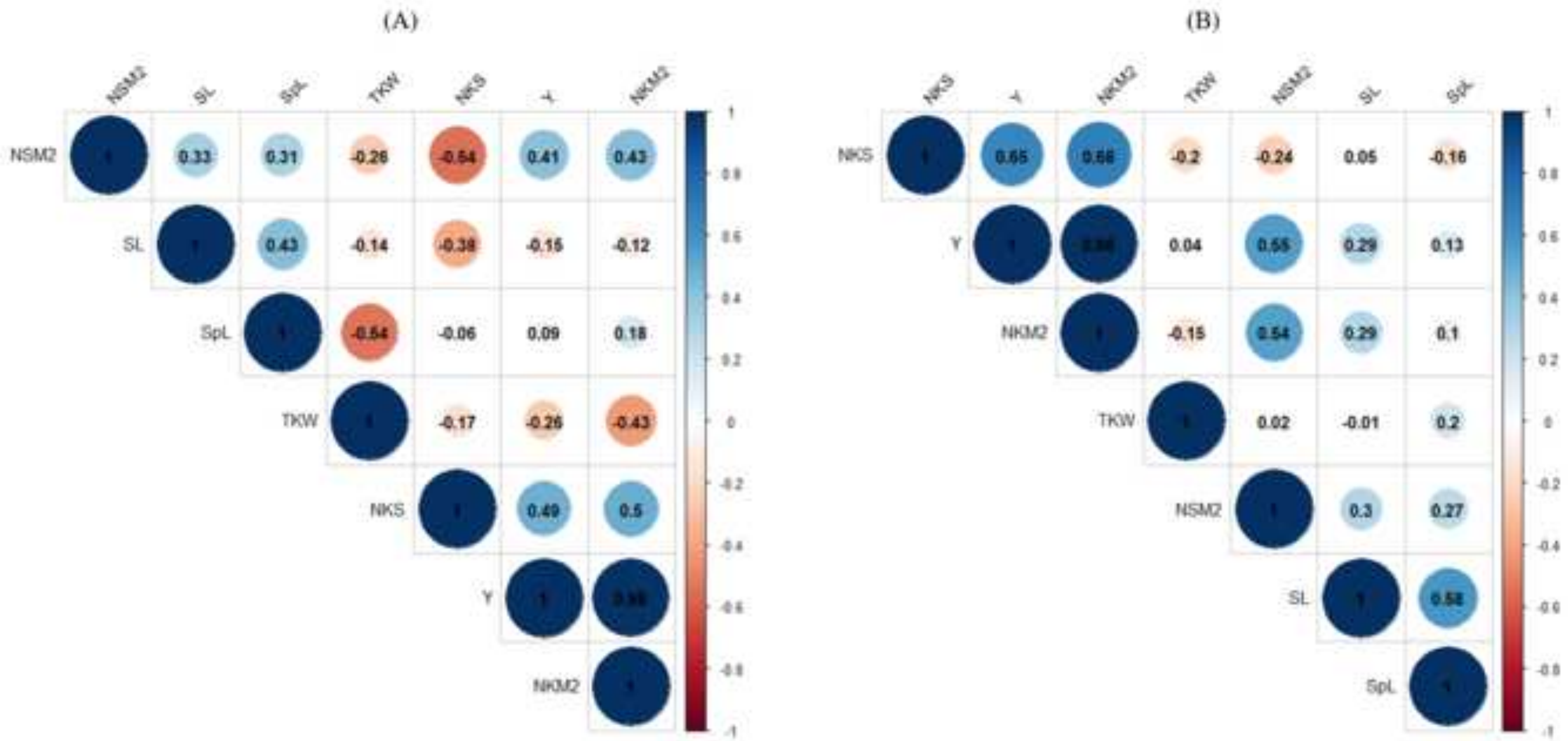

Click here to access/download;Figure;Fig 3.tif

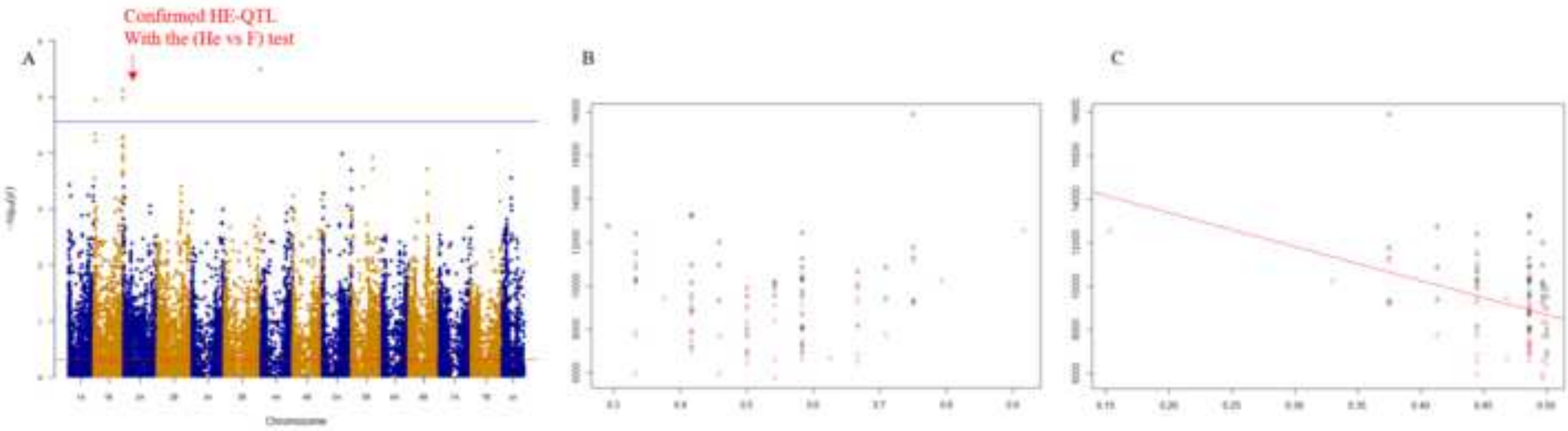

Click here to access/download;Figure;Fig 4.tif

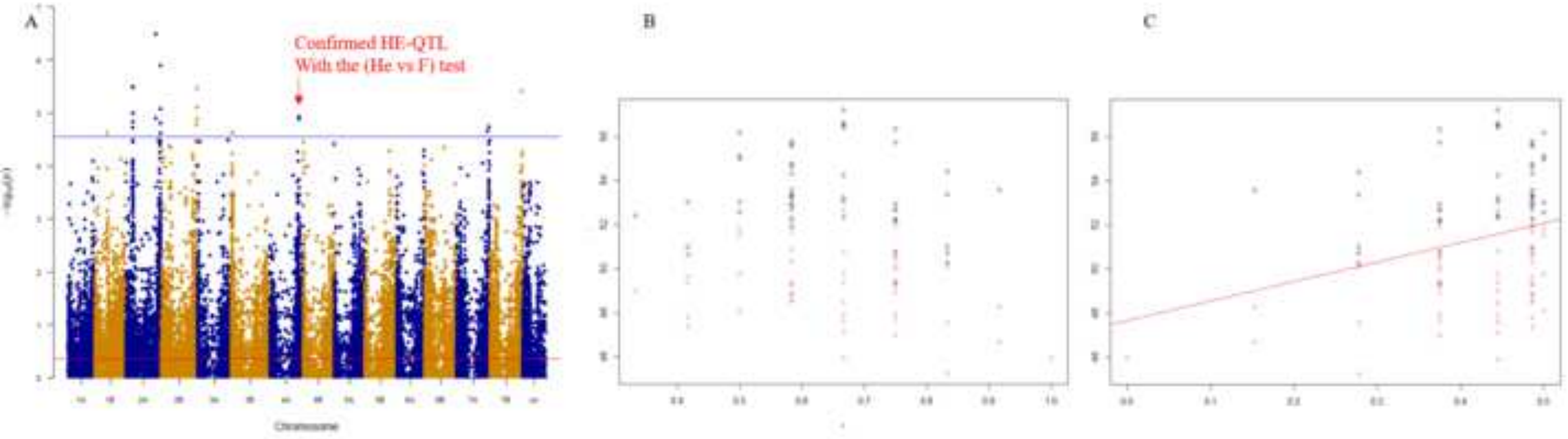

Click here to access/download;Figure;Fig 5.tif

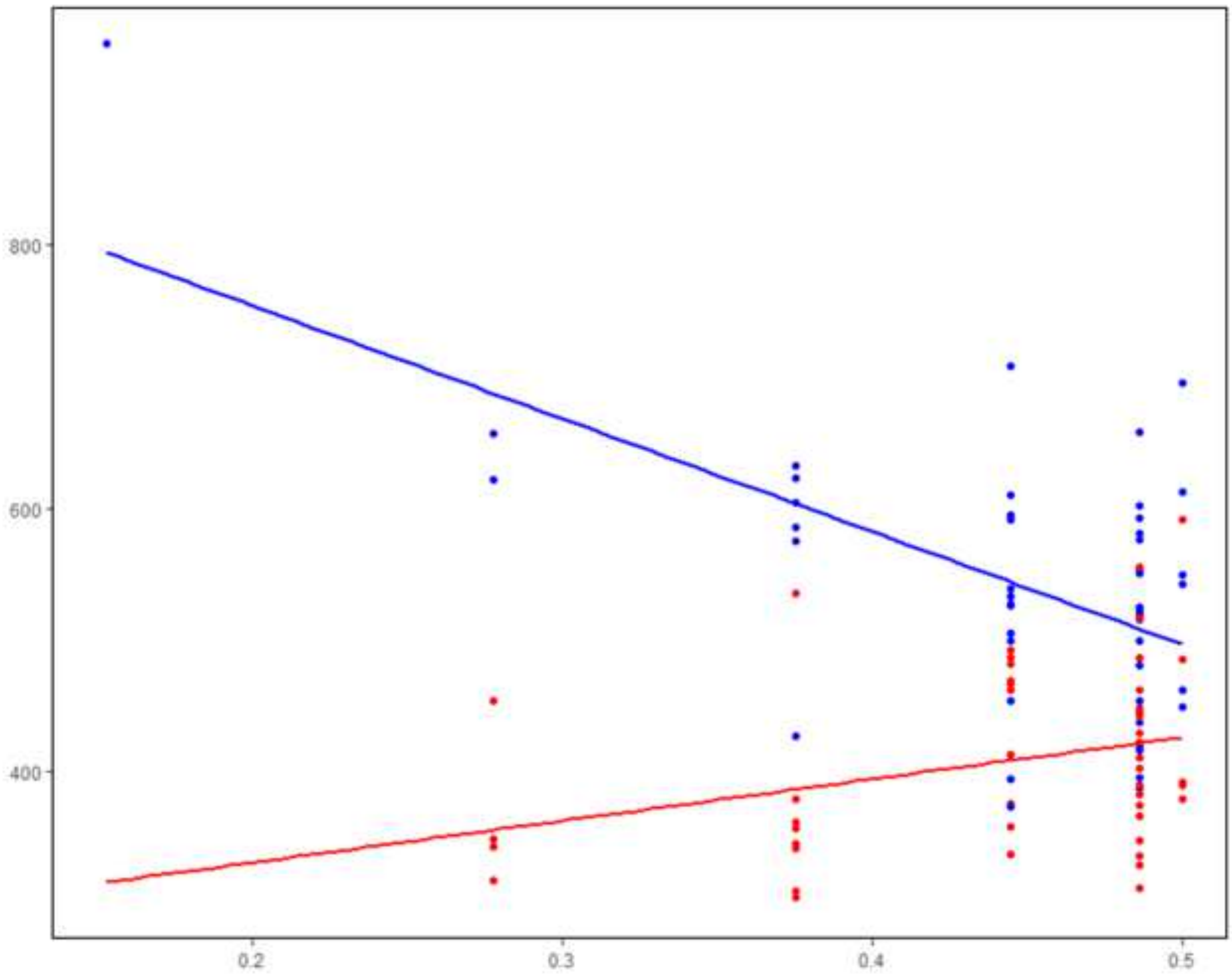

Click here to access/download;Figure;Fig 6.tif

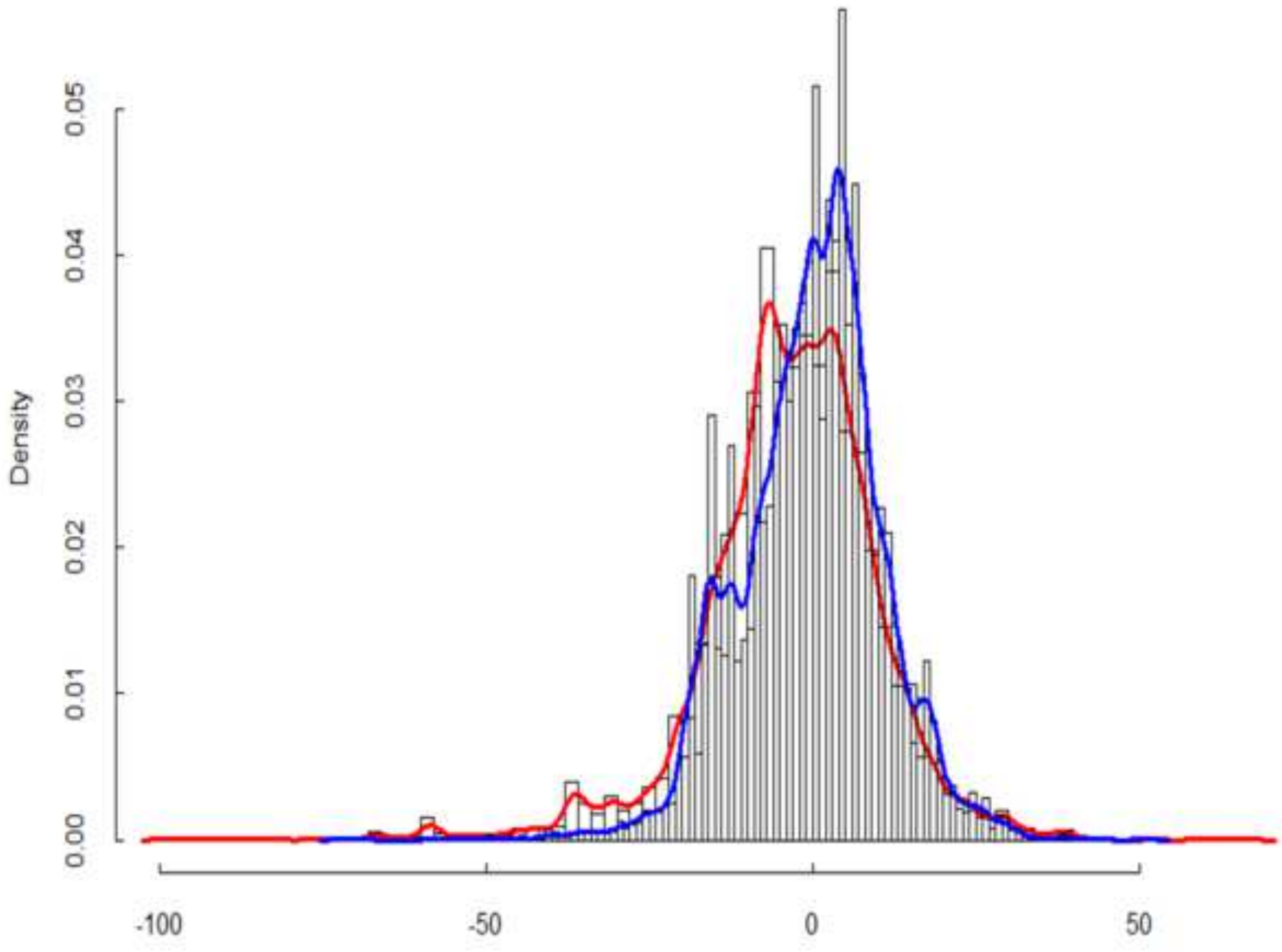

Click here to access/download
Supporting Information
Alsabbagh_Supplementary_Information_11022021.docx

Click here to access/download
Supporting Information
S1 Fig.tif

Click here to access/download
Supporting Information
S2 Fig.tif

Click here to access/download
Supporting Information
S3 Fig.tif
