# Supplementary material for: Diversity matters in wheat mixtures: A genomic survey of the impact of genetic diversity on the performance of 12 way durum wheat mixtures grown in two contrasted and controlled environments
Source: PLoS One. 2022 Dec 9;17(12):e0276223. doi: 10.1371/journal.pone.0276223 (PMC9733896; doi:10.1371/journal.pone.0276223)
Supplement: Attachment — Submitted filename: Response to reviewers.docx [file pone.0276223.s009.docx]

Revised Article: *(Highlighted in yellow in the “Revised Manuscript with Track Changes”)*

We revised the manuscript of the article and made improved the text based on the comments of the two reviewers.

Note that we have added two new authors: Germain Montazeaud and Morgane Ardisson.

We have improved the figures: in figure S1, S2 and 1 we deleted the legend from the figures and keep it just in the title of the figures. In figure S3, 3, 4 and 5 we improved the quality and changed the colours and legends of the figures. In figure 2 we regrouped the 2 figures we had in one clearer figure following the reviewer's request.

We have improved the turns of the sentences, added new references and improved the bibliography.

We changed the sentence explaining where the funding comes from, according to the funders’ demand.

Here are the detailed corrections below:

Funding:

“This project has received funding from the European Union’s Horizon 2020 research and innovation programme under grant agreement N°727247”.

PA recieved a grant from "l'Association de spécialisation et d'orientation scientifique, Liban".”

"The funders had no role in study design, data collection and analysis, decision to publish, or preparation of the manuscript."

Abstract:

**Line 42:** “the greater the unfavourable conditions” instead of “the greater the stress”.

Introduction:

**Line 51:** It was proposed to change of “increased climate variability induced by climate change” by “increased climate change induced by climate variability”. We kept our intitial sentence.

**Line 57:** “natural fields” instead of “real fields”.

**Line 59:** “the” instead of “this”.

**Line 60:** I deleted “both” and I changed “field” into “fields”.

**Line 61:** “was” instead of “is indeed”.

**Line 66:** “in” instead of “for”.

**Line 89:** “according to the environmental stress” changed in “because of”

**Line 92:** “Under this assumption, species redundancy in diverse communities are able to maintain functioning during increased environmental stress. Tolerant species within the mixture provides a greater guarantee to maintain functioning by compensating the failure of sensitive species.” Instead of “Under this assumption, diverse communities are able to maintain functioning during increased environmental stress due to species redundancy that provides a greater guarantee to maintain functioning due to the tolerant species within the mixture, compensating for the failure of sensitive species.”

**Line 95:** I deleted “The intensity of”. So the sentence is “Facilitation and competition between plants varies along environmental gradients with clear positive interactions dominating under stressful conditions.” Instead of “The intensity of facilitation and competition between plants thus varies along environmental gradients with clear positive interactions dominating under stressful conditions.”

**Line 116:** “Majority of the studies tackling varietal mixtures, as these two studies are based on two-way balanced mixtures.” instead of “These two studies, like the vast majority of the studies that tackle the question of varietal mixtures, are based on two-way balanced mixtures.”

Materials and methods:

**Line 142:** deleted the double [49–51].

**Line 144, 145, 146:** “We discarded extreme tall and short components to reduce the competition among lines within plots, due to plant height” instead of “We discarded extreme tall and short components to reduce the impact of an obvious and already well-described competition due to plant height for light and resources among lines within plots.”

**Line 147:** I deleted “extracted from EPO”.

**Line 150:** I deleted “(96)”.

**Line 150:** “… for each of them a number of 70 seeds from twelve distinct durum wheat lines, …” instead of “… for each of them an even of seeds (70) from twelve distinct durum wheat lines,..”

**Line 180:** “four depths” instead of “four depth”.

**Line 194:** “seedburo” instead of “Seedburo”.

**Line 207:** we added “using homemade scripts”.

**Line 209:** we added **“**with a minimum of 95% similarity”.

**Line 228 – 231**: we clarified as follows

We kept the 31,059 SNPs whose minimum frequency in the mixtures was below 0.5 and for which the maximum frequency was above 0.5. In addition, we imposed that the difference between these minimum and maximum values be at least 0.5

**Line 237:** “was analysed” instead of “was realized”.

**Line 243:** We precised

“The genome wide diversity association (GWDA), respectively the genome wide frequency association (GWFA).”

**Line 243:** “… on the diversity index, HE, resp, the allelic frequency F, …” instead of “… on the genetic diversity HE, resp, the allelic frequency F …”.

**Line 244:** We rephrase and simplify the sentences introducing the model. We also declared the water regime effect and its interactions with both genetic variables (F and HE).

**Line 273:** There is no a specific name for the method used by [58]. They proposed a method since there is no widespread method to define QTL boundaries from GWAS results. We just added “A linkage disequilibrium based method”.

Results:

**Line 344:** We changed the figure 2 and then its title and legend. We wrote “Pairwise Pearson correlation coefficients (r) between functional traits for the 48 mixtures under controlled water deficit conditions (above the median) and the 48 mixtures under rain-fed conditions (below the median). Positive correlations are in blue and negative in red. The darker and larger the square, the higher the correlation between traits. Traits have been clustered according to the *hclus* procedure of the *corrplot* R package function.” instead of “ Pairwise Pearson correlation coefficients (r) between functional traits for all the 48 mixtures (A) under controlled water deficit conditions and (B) the 48 mixtures under rain-fed conditions. The significant threshold has been set at 5%. Positive correlation are in blue and negative in red. More the circle is bi 347 g more the correlation is significant and vice versa. More the colour of the circle on the number is dark more the correlation is big and vice versa. Traits have been clustered according to the hclus procedure of the corrplot R package function.”

**Line 400:** We changed the figure 3 and then its title and legend. We wrote “Association between the number of kernels per m² (NKM2) and genomic diversity. (A) Manhattan plot reporting p-values (-log10 transformed) for the association tests between NKM2 and diversity index (Nei’s diversity) at 31k eligible SNPs distributed along the durum wheat genome (GWDA). The solid blue line represents the Family-Wise Error Rate (FWER) of 5% computed with the Galwey method. The SNP “AX-89411835” located on the chromosome 1B has passed the He vs. F test (see text). (B) Association between of the 96 allelic frequencies of the SNP “AX-89411835” in the mixtures and their NKM2 values. Red points correspond to control water deficit plots, blue points to rain-fed plots. (C) Relationships between the genetic diversity of the SNP “AX-89411835” and NKM2. Lines are the slopes of regression. The slope for the controlled water deficit treatment has been computed without the outlier point on the x-axis.” instead of “Negative impact on NKM2 of genetic diversity of the SNP “AX-89411835” located on the chromosome 1B. (A) Manhattan plot reporting p-values (-log10 transformed) for the association tests between NKM2 and diversity at 31k SNPs distributed along the durum wheat genome (GWDA). The solid blue line represents the Family-Wise Error Rate (FWER) of 5% computed with the Galwey method. (B) Distribution of the allelic frequency of the SNP “AX-89411835” located on the chromosome 1B and NKM2. Red points corresponds to stressed plots. Black points corresponds to rain-fed plots. (C) Relation between the genetic diversity of the SNP “AX-89411835” and NKM2. Red points corresponds to stressed plots. Black points corresponds to rain-fed plots. The red line represents the slope of the regression.”

**Line 409:** We changed the figure 4 and then its title and legend. We wrote “Positive impact on thousand-kernel weight (TKW) of the genetic diversity at the SNP “AX-89377854” located on the chromosome 4A. (A) Manhattan plot reporting p-values (-log10 transformed) for the association tests between TKW and diversity at 31k eligible SNPs distributed along the durum wheat genome (GWDA). The solid blue line represents the Family-Wise Error Rate (FWER) of 5% computed with the Galwey method. (B) Association between the allelic frequency of the SNP “AX-89377854” and TKW. Red points corresponds to plots under controlled water deficit conditions. Blue points corresponds to plots under rain-fed conditions (C) Relation between the genetic diversity of the SNP “AX-89377854” and TKW. Lines represent the slopes of the regression.” instead of “Positive impact on thousand-kernel weight (TKW) 409 of the genetic diversity of the associated SNP “AX-89377854” located on the chromosome 4A. (A) Manhattan plot reporting p-values (-log10 transformed) for the association tests between TKW and diversity at 31k SNPs distributed along the durum wheat genome (GWDA). The solid blue line represents the Family-Wise Error Rate (FWER) of 5% computed with the Galwey method. (B) Distribution of the allelic frequency of the SNP “AX-89377854” and TKW. Red points corresponds to plots under controlled water deficit conditions. Black points corresponds to plots under rain-fed conditions (C) Relation between the genetic diversity of the SNP “AX-89377854” and TKW. Red points corresponds to plots under controlled water deficit conditions. Black points corresponds to plots under rain-fed conditions. The red line represents the slope of the regression.”

**Line 445:** We changed the figure 5 and then its title and legend. We wrote “Contrasted impact of the genetic diversity at locus “AX-89687986” on the yield (Y) on the 96 durum wheat mixtures under two water regimes: in red, positive effect of diversity under controlled water deficit conditions (red points and line) and in blue negative effect (ns) under Rain-fed conditions.” instead of “Contrasted impact of the genetic diversity at locus “AX-89687986” on Y under two water regimes: positive effect of diversity under controlled water deficit conditions (red points and line) and negative effect (ns) under Rain-fed conditions (blue points and line).”

Discussion:

**Line 524:** I deleted the title.

**Line 547:** We clarified as follows

The diversity effects at the genomic level have been reported previously [47,52], in experiments in which neighbouring genotypes were grown in spatially alternative rows. Here, we report on a completely random neighbourhood among genotypes in highly complex mixtures.

**Line 556:** “The regression approach used in GWAS analysis is sensitive …” instead of “As any GWAS analysis, the regression approach used is sensitive to…”.

**Line 559:** “as it” instead of “since it”.

**Line 559:** After discussions with a statistician, we change the text and propose to explore in a future work the non linearity between plant x plant interaction and diversity. To our knowledge, this has not been done yet. We added :

“If the GWDA is regressing on one predictor (HE), as in GWFA (F), it is not yet clear if statistical bias could generate an excess of false associations or if it better take into account plant x plant interactions in a heterogeneous population since it permits to fit non-linear relationships between allele frequencies and the trait. Investigation using simulations would be necessary”

**Line 707:** “her” instead of “their”.

**Line 708:** “due to lack of sufficient studies” instead of “due to a lack of information (not enough studies and not enough details about the nature of the stress)”.

**Correction based on reviewers notes:**

Reviewer #2: *(Highlighted in blue in the “Revised Manuscript with Track Changes”)*

We only consider here the changes that are specific to reviewer2 since they had common ones

**Line 192:** (L.192 – L. 196): “The whole plot harvest was divided by half with a seedburo divider followed by second division by 8 using a precision Retsch divider. A final count of 1200 kernels from a sample was done on a Contador device (Pfeuffe) and then precisely weighed. This is how we obtained a representative sampling of 1200 kernels from each plot.”

Instead of “The thousand Kernels Weight (TKW) was obtained from a representative sampling of 1200 kernels from the whole plot harvest after first division by half with a seedburo divider followed by second division by 8 using a precision Retsch divider. A final count of 1200 kernels was done on a Contador device (Pfeuffe) and then precisely weighed.”

**Line 243:** “were built” instead of “were build”.

**Line 338:** “For instance, plots having longer stem were more productive in the R condition (r(SL-NKM2)=+0.3, p-value= 0.03) but under CWD we didn’t find significant correlation between stem length and productivity (-0.12, p-val=0.4).”

Instead of “For instance, plots having longer stem were more productive in the R condition (r(SL-NKM2)=+0.3, p-value= 0.03) but with no significant correlation under CWD (-0.12, p-val=0.4).”

**Line 546:** “These approaches, as the genome-environment association (GEA) analysis often use the Pool-Seq approach in the aim to uncover SNPs associated to climatic or plant community variation [81–83].” **i**nstead of “These approaches, as the genome-environment association (GEA) analysis often combine a Pool-Seq approach in the aim to uncover SNPs associated to climatic or plant community variation [78–80].”

**Line 620:** “Yes” if the number of SNPs that have positive impact of their diversity on the traits under CWD is significantly more than the number under R, … instead of “Yes” if a significantly more SNP had a more positive impact of their diversity under CWD than under R, …

**Line 661:** “affect” instead of “complexity”.

**Line 577:** “we confirm” instead of “we believe”.

**Line 311:** “The soil water reserve in the R plots was never significantly” instead of “The soil water reserve in the R plots never felt significantly”

**Line 598:** “what they could have detect” instead of “what they could have felt”.

**Line 584:** “shows” instead of “proves”.

**Line 294:** “31,059” instead of “13,093”.

Funding information:

**Line 724:** we wrote “This project has received funding from the European Union’s Horizon 2020 research and innovation programme under grant agreement N°727247” instead of “The EU H2020 SOLACE funded the experiment”.
